# The relationship between ionic-electronic coupling and transport in organic mixed conductors

**DOI:** 10.1126/sciadv.adi3536

**Published:** 2023-08-30

**Authors:** Scott T. Keene, Akshay Rao, George G. Malliaras

**Affiliations:** ^1^ Department of Engineering, Electrical Engineering Division, University of Cambridge, Cambridge, CB3 0FA, UK.; ^2^ Cavendish Laboratory, Department of Physics, University of Cambridge, Cambridge, CB3 0HE, UK.

## Abstract

Organic mixed ionic-electronic conductors (OMIECs) directly convert between ionic and electronic charge through electrochemical (de)doping, enabling a wide range of applications in bioelectronics, neuromorphic computing, and energy storage and conversion. While both ionic and electronic transport are individually well characterized, their combined transport has been difficult to describe self-consistently. We use in situ measurements of electrochemical (de)doping of an archetypal OMIEC to inform a quasi-field drift-diffusion model, which accurately captures experimentally measured ion transport across a range of potentials. We find that the chemical potential of holes, which is modulated by changes in doping level, represents a major driving force for mixed charge transport. Using numerical simulations at device-relevant time scales and potentials, we find that the competition between hole drift and diffusion leads to diffuse space charge regions despite high charge densities. This effect is unique to mixed conducting systems where mobile ionic charges can compensate the accumulation or depletion of electronic charge, thereby screening electrostatic driving forces.

## Teaser

The hole chemical potential drives diffusive kinetics for electrochemical doping in an ion conducting conjugated polymer.

## INTRODUCTION

Materials that conduct both electronic and ionic charges through the bulk of their volume are crucial for emerging electrochemical devices (*1*). Among this materials class, organic mixed ionic-electronic conductors (OMIECs) have emerged as a highly promising candidate due to their biocompatibility and relatively high free volume, which facilitates the uptake of bulky ionic species without undergoing damaging strains. For this reason, OMIECs have been adopted as a choice material for applications in bioelectronics (*2*), chemical sensors (*3*, *4*), neuromorphic computing and memory (*5*, *6*), and energy conversion and storage (*7*).

While widely used, there still exist many inconsistencies across descriptions of the underlying OMIEC device physics. One of the defining features of OMIECs is their volumetric capacitance (*C^∗^*), which results from ion penetration into the bulk of the material, either displacing (dedoping) or compensating (doping) electronic charge on the semiconducting polymer backbone (*8*, *9*). For this reason, most organic electrochemical transistor (OECT) device models start with a capacitive description introduced by Bernards and Malliaras (*10*), which uses *C^∗^* to determine the charge carrier concentration by calculating the local electric potential. Then, the steady-state carrier distribution and channel current are solved for using Ohm’s law and Kirchhoff’s law along the length of the channel. Friedlein *et al.* (*11*) expanded on this model to account for the carrier concentration–dependent mobility of holes in the OMIEC channel to better match experimental results.

Recently noted by Kaphle *et al.* (*12*), the ionic and electric potential distributions predicted by capacitive models are unphysical as they would be expected to drive lateral ionic fluxes along the channel. Rather than using *C^∗^* as the governing equation, Kaphle *et al.* (*12*) used a drift-diffusion model to describe the steady-state OECT characteristics, yielding an exponential distribution of carriers along the channel length. However, such electrostatic drift-diffusion models do not capture the volumetrically capacitive behavior. Tybrandt *et al.* (*13*) have similarly used a drift-diffusion model to describe behavior for several OMIEC-based devices, which includes the hole chemical potential of holes near the tail of the density of states (DOS). In this model, *C^∗^* is accounted for by defining and independently solving Poisson’s equation for two separate electric potentials corresponding to the ionic and electronic phases where the interface between the phases is treated as a double-layer capacitor. However, in a wide range of OMIECs, there are not distinct ionic and electronic phases, with many single-component polymers (*14*), and in blends such as poly(3,4-ethylenedioxythiophene):poly(styrenesulfonate) (PEDOT:PSS), the phases lack discrete boundaries (*15*, *16*). While a wide range of drift-diffusion models have been applied to OMIECs (*17*), none have emerged as a replacement to the commonly used Bernards model (*10*).

**Fig. 1.**
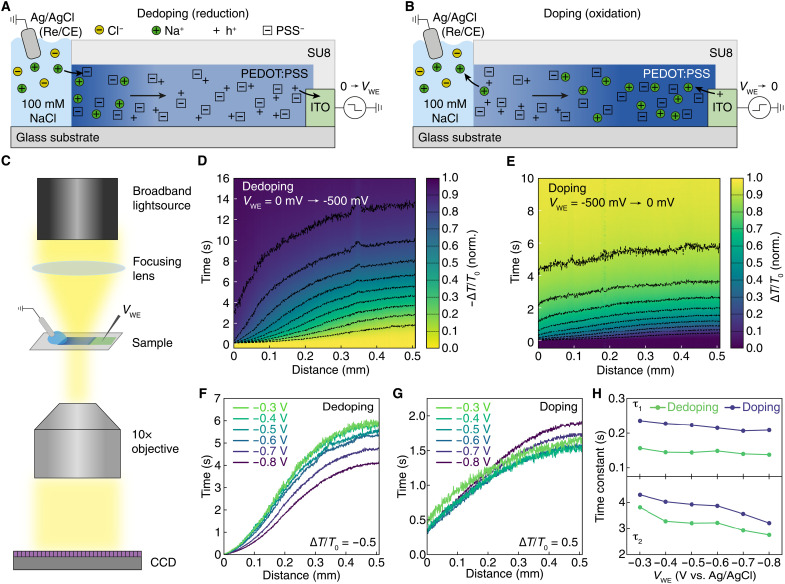
Optical measurements of (de)doping kinetics in PEDOT:PSS. Schematic of propagation of electrochromic transition during electrochemical (**A**) dedoping and (**B**) doping of PEDOT:PSS. (**C**) Experimental setup for optical tracking of ions through a PEDOT:PSS thin film. CCD, charge-coupled device. Normalized differential transmission (∆*T*/*T*_0_) through a PEDOT:PSS film during (**D**) dedoping and (**E**) doping with an applied bias of *V*_WE_ = −500 mV. Dashed black contour lines mark crossing incremental ∆*T*/*T*_0_ values (from 0.1 to 0.9, increments of 0.1). Transmission intensities are measured over the spectral region from 1.5 to 2.6 eV where absorption is dominated by neutral PEDOT chains. Voltage dependence of ion transport using ∆*T*/*T*_0_ = 0.5 as a reference for (**F**) dedoping and (**G**) doping. (**H**) Time constants τ_1_ and τ_2_ for (dis)charging current (*I*_WE_) decay during dedoping (green) and doping (purple).

In this work, we build off the work of Tybrandt *et al.* (*13*) to introduce a mixed ionic-electronic transport model describing electrochemical (de)doping kinetics, which is compatible with both electrodynamics and volumetric capacitance by including the hole chemical potential as a driving force for hole transport. Since the seminal work by Kroemer (*18*), it has been well understood that gradients in the chemical potential, or the quasi-Fermi level of carriers, can effectively act as a carrier-specific electric field, often referred to as a quasi-electric field. However, this effect is seldom accounted for in models describing mixed ionic-electronic transport despite the large changes in hole chemical potential associated with electrochemical (de)doping (*19*). Here, we develop a drift-diffusion model that accounts for quasi-Fermi level gradients, which drive hole transport to accurately describe experimental characterization of ion motion during both dedoping (Fig. 1A) and doping (Fig. 1B) of the prototypal OMIEC, PEDOT:PSS, at device-relevant potentials.

The results show that, while initial ionic and electronic currents are dominated by drift, hole quasi-electric fields drive large diffusion currents, which broaden the internal electric fields and alter the kinetics of electrochemical (de)doping. We also find that the motion of holes under the applied field leaves net charge imbalance leading to ion fluxes, which follow behind holes and are dominated by drift. This leader-follower relationship between holes and ions results in (de)doping speeds that greatly exceeds those expected flux for ion diffusion alone, with the experimental (de)doping speeds approaching the expected value for ion drift-limited doping (*20*–*22*). Last, we find that steady state occurs when both the hole and ion electrochemical potentials are uniform across the OMIEC-electrode and OMIEC-electrolyte interfaces, respectively.

The conclusions of this work question the applicability of device models that presume that electronic currents in OMIECs are dominated by hole drift under an electric field (*10*). Instead, the internal electric fields in operating OMIEC-based devices are likely small with diffusion driven by quasi-electric fields playing a large role in both the transient response and steady-state characteristics of OECTs. Diffusion has been shown to constitute a major driving force in other mixed ionic-electronic devices, namely, light-emitting electrochemical cells (*23*–*25*) where the initial displacement of ions results from drift, but the steady-state behavior is dominated by the diffusion of injected carriers. Last, the mixed ionic-electronic transport model presented here is unlikely to be unique to organic systems, extending to other mixed conducting systems that undergo large modulation in electronic carrier densities during operation.

## RESULTS

### Microscopic measurements of ion transport

We use optical transmission microscopy to monitor ion motion through a ca. 225-nm-thick, 500-μm-long PEDOT:PSS film (Fig. 1C) during electrochemical dedoping and doping. The decrease (increase) in transmission (∆*T*/*T*_0_) is due to an increase (decrease) in the absorption of neutral PEDOT chains as PEDOT is dedoped (doped) (*15*, *20*, *26*, *27*). The result for dedoping of PEDOT:PSS (*V*_WE_ from 0 to −500 mV versus Ag/AgCl) is a sigmoidal shape where dedoping happens rapidly at the OMIEC-electrolyte interface, then broadens with increasing distance along the length of the film (Fig. 1D). To visualize the dedoping kinetics, we normalize ∆*T*/*T*_0_ between 0 and −1, where 0 is the transmission intensity before a potential is applied, and −1 is the final transmission after the dedoping is complete. The broadening of the dedoping front can be observed as the increasing temporal separation and flattening of the dashed contour lines, which highlight isointensity profiles, or transitions across distinct doping levels.

The subsequent doping of PEDOT:PSS from the dedoped state (*V*_WE_ from −500 to 0 mV versus Ag/AgCl) shows an increase ∆*T*/*T*_0_ (normalized from 0 to 1), which moves from the electrolyte to the indium tin oxide (ITO) contact (Fig. 1E). In contrast to dedoping, the isointensity contour lines show that the doping profile is broad along the entire length of the PEDOT:PSS film, and the overall time scale of doping is faster than dedoping, consistent with previous reports (*28*, *29*).

Following previous work (*15*, *20*), we use the transition across the halfway point between the initial and final transmission (i.e., |∆*T*/*T*_0_| = 0.5) (Fig. 1, F and G) to compare (de)doping speeds across a range of driving potentials, where ∆*T*/*T*_0_ is normalized between 0 and ±1 for each measurement to better compare across potentials, which yield different final (initial) doping levels. In contrast to previous models (*30*), the dedoping time shows a weak dependence on potential, with the curves corresponding to *V*_WE_ = −0.3 to −0.6 V versus Ag/AgCl overlapping. At higher applied potentials (−0.7 and −0.8 V versus Ag/AgCl), the dedoping fronts become moderately faster. The doping fronts have a flatter profile than dedoping fronts and occur over shorter time scales, also showing a weak time dependence when changing the initial applied potential (Fig. 1G). We also compare the optically measured (de)doping times to the time constants (τ) obtained from the chronoamperometry current using a commonly used biexponential decay (*31*, *32*) (Eq. 1).

$$I(t)=I_{0}-I_{1}\left\{1-\exp\left(\frac{t}{\tau_{1}}\right)\right\}-I_{2}\left\{1-\exp\left(\frac{t}{\tau_{2}}\right)\right\}$$ (1)

where *I*_0_, *I*_1_, and *I*_2_ are constants describing the magnitude of current and τ_1_ and τ_2_ are the characteristic time scales for dedoping (doping) are plotted as a function of the final (initial) *V*_WE_. The full fitting results can be found in table S1. The τ_1_ and τ_2_ values show a similar lack of dependence on the applied potential and, in contrast to the optical data, show a faster dedoping kinetic compared to doping (Fig. 1H), confirming that the time scale for (de)doping of PEDOT:PSS is only weakly dependent on the applied potential.

### Quantifying local hole concentrations

**Fig. 2.**
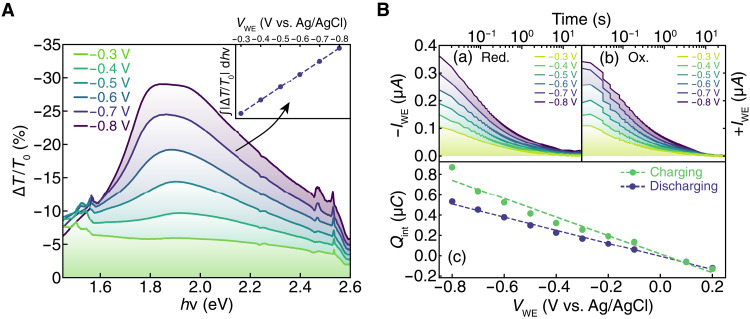
Quantifying local hole concentrations. (**A**) Differential transmission spectra ∆*T*/*T*_0_ for (de)doping across a range of potentials. The absolute value of the integral of the spectra plotted against the potential (inset) shows a linear relationship between transmission intensity and applied potential. (**B**) Chronoamperometry from reduction (a) and oxidation (b) experiments at different potentials. (c) Integrated charge extracted during charging and injected during discharging for experiments at varied potentials showing a linear dependence between charge and the applied potential.

The relative transmission intensity through PEDOT:PSS in the visible range (1.6 to 2.5 eV) is expected to be related to the concentration of absorbing neutral PEDOT chains (*28*, *33*). Thus, ∆*T*/*T*_0_ images should provide quantitative information about the local change in hole concentration, *p*. To quantify the relationship between ∆*T*/*T*_0_ and *p*, we measured the voltage-dependent transmission spectra (Fig. 2A), which shows decreased transmission at negative potentials, consistent with increasing neutral PEDOT chain absorption as PEDOT:PSS is further dedoped. The differential transmitted intensity (∆*T*/*T*_0_) is calculated with respect to the transmitted intensity at the beginning of the measurement (*T*_0_, *V*_WE_ = 0 V versus Ag/AgCl), where ∆*T* is given by *T*_f_ − *T*_0_, where *T*_f_ is the average transmitted intensity across the entire sample at the end of (de)doping (*t* = 30 s). By taking the integral of ∆*T*/*T*_0_ across the lamp spectrum at each voltage, we find a linear relationship between ∆*T*/*T*_0_ and the applied potential (Fig. 2A, inset).

To verify that the typical volumetric capacitance is preserved for PEDOT:PSS in the moving front sample geometry, we integrated the chronoamperometry current transients as a function of voltage for both charging and discharging (Fig. 2B). We find a linear relationship between the applied voltage and total extracted/injected charge (∆*Q*), indicating that the confined PEDOT:PSS charges capacitively and that *C^∗^* is constant over the experimental voltage range. The discrepancy between charging and discharging is likely due to the presence of oxygen, which can be reduced by PEDOT:PSS at negative potentials (*34*). The linear relationship between ∆*T*/*T*_0_ and potential and between ∆*Q* and potential allows us to relate ∆*T*/*T*_0_ to the local changes in carrier density, ∆*p*(*x*), given in Eq. 2 below.

$$\frac{\text{Δ}T}{T_{0}}(x)=\text{Δ}p(x)\frac{e}{C^{*}}S$$ (2)

where *S* is the slope of the curve in the inset of Fig. 2A. Using Eq. 2, we infer that ∆*T*/*T*_0_ maps in Fig. 1 (D and E) are proportional to the time-dependent local hole concentrations during (de)doping. A detailed discussion of the experimental conditions necessary for Eq. 2 to remain valid is included in the Supplementary Text. For the high changes in carrier densities of (de)doping experiments, electroneutrality can only be broken at very small length scales (i.e., at the Debye length), allowing quantification of the local ion concentrations in the film as well. The quantitative measures of hole and ion concentrations help understand the (de)doping process phenomenologically as discussed in the next section.

### Mechanism of (de)doping of PEDOT:PSS

**Fig. 3.**
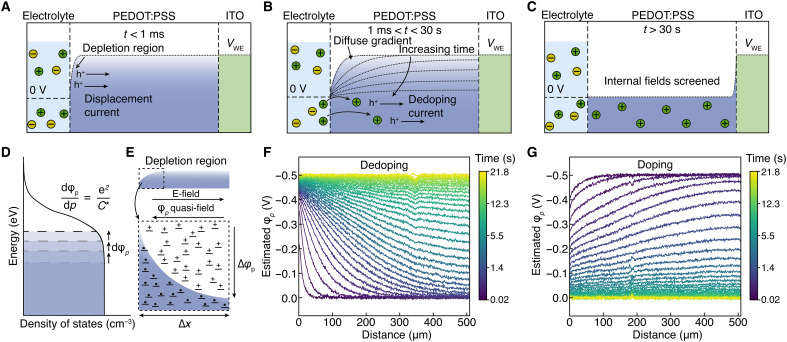
Driving forces for electrochemical (de)doping of PEDOT:PSS. Schematic description of the dedoping process in PEDOT:PSS. (**A**) When a potential is applied to the ITO contact, there is small hole displacement current to equilibrate the potential across the ITO/PEDOT:PSS, leaving a hole depletion region at the electrolyte interface. (**B**) Cations drift into PEDOT:PSS due to the electric field, dedoping PEDOT. This ion displacement leads to a decrease in the local electric potential. Rather than forming a sharp boundary between the dedoped and doped regions, the potential gradients become broad. (**C**) Dedoping finishes when the internal potential reaches the potential in the electrolyte, leaving a flat potential landscape for holes and cations. The final hole concentration will depend on the (**D**) hole chemical potential (ϕ*_p_*) shift during dedoping such that there is no driving force for hole transport across the PEDOT:PSS/ITO interface. (**E**) The concentration-dependent hole chemical potential results in a hole quasi-electric field driving diffusion in the opposite direction of drift resulting in diffuse potential gradients. Estimated evolution of the hole chemical potential profile from experimental data during (**F**) dedoping (*V*_WE_ from 0 to −0.5 V versus Ag/AgCl) and (**G**) doping (*V*_WE_ from 0 to −0.5 V versus Ag/AgCl). The color of each trace in (F) and (G) indicates the time after applying *V*_WE_ as described by the color bar, where the temporal spacing between successive traces increases linearly from the first time increment of 20 ms (purple curve) to 1.3 s (yellow curve).

When *V*_WE_ is applied to the ITO, the electric potential within the highly conductive PEDOT:PSS should rapidly reach *V*_WE_ via a hole displacement current, analogous to a metal-oxide-semiconductor capacitor. Since the electrolyte is held at 0 V, a depletion region, and therefore electric field, will form in PEDOT:PSS near the electrolyte interface (Fig. 3A). Then, because cations are free to move across this interface, they will drift into the PEDOT:PSS, resulting in dedoping near the electrolyte interface (Fig. 3B). The movement of ions alters the charge density in the depletion region, leading to a change in the electric potential profile. Rather than maintaining a sharp boundary between the dedoped and doped regions of the film as previously predicted (*30*), dedoping occurs relatively evenly along the length of the film, resembling a diffusion-limited transport (see simulations in fig. S1). Dedoping then finishes when there are no remaining driving forces for further ion or hole currents (i.e., when the internal electric field goes to 0) (Fig. 3C).

At steady state, there must not be any driving forces for hole or ion motion. Within the PEDOT:PSS film, this requirement is satisfied by the lack of internal electric fields or ion or hole concentration gradients. However, the electric potential difference between the ITO contact and PEDOT:PSS should drive a hole current. This inconsistency can be resolved by considering the origin of the volumetric capacitance. The scaling of charge storage capacity with volume indicates that the doping is related to the thermodynamic potentials of holes and ions rather than the penetration depth of the electric field as is the case for field-effect devices. For this reason, volumetric capacitance is often described as the change in chemical potential (i.e., quasi-Fermi level) (ϕ*_p_*) of electrons or holes as a function of changes in their concentration (*19*) described in Eq. 3 below and schematically depicted in Fig. 3D.

$$d\varphi_{p}=dp\frac{e}{C^{*}}$$ (3)

This means that, at steady state, ϕ*_p_* must equal the electric potential in the ITO contact such that there is no electrochemical potential gradient to drive hole current across the interface. Since *C*^∗^ is constant across the experimental conditions, we can relate the local hole chemical potential to the hole concentration as described in Eq. 4 below.

$$\varphi_{p}(x)=\frac{e}{C^{*}}[p(x)+p_{0}]$$ (4)

where *p*_0_ is a reference hole concentration where the chemical potential of holes is equal to the electrode potential at 0 V. Last, using Eq. 4, we can relate the optical transmission profiles to the hole chemical potential as described in Eq. 5.

$$\frac{\text{Δ}T}{T_{0}}(x)\propto \varphi_{p}(x)$$ (5)

Using Eq. 5, we can estimate the gradients in hole chemical potential, which act as a quasi-electric field (*18*) driving hole transport during dedoping (Fig. 3F) and doping (Fig. 3G). We use the initial and final potential at the ITO contact of 0.0 and −0.5 V versus Ag/AgCl, respectively, to calibrate the differential transmission to the chemical potential of holes relative to the contact.

### Drift-diffusion simulations with hole quasi-electric fields

**Fig. 4.**
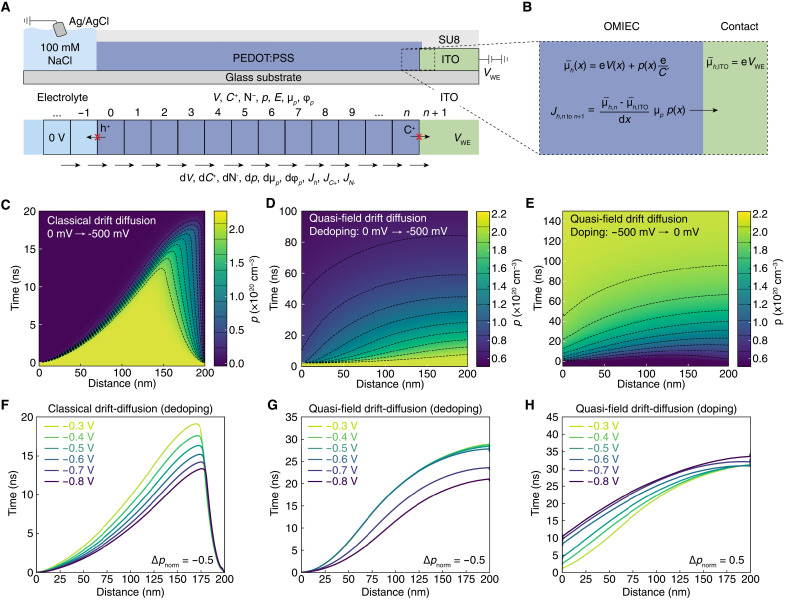
Drift-diffusion modeling of (de)doping in OMIECs. (**A**) Schematic showing finite elements simulation procedure where intensive quantities are calculated at each length increment and the gradients are calculated across the boundaries of length increments. (**B**) Flux across the OMIEC-electrode interface is given by the difference in electrochemical potential of holes. (**C**) Simulation results using the classical drift-diffusion model during dedoping (*V*_WE_ from 0 to −500 mV versus Ag/AgCl) showing a sharp transition between doped and undoped parts of the film. Simulation results when incorporating hole quasi-electric fields into the drift-diffusion model for (**D**) dedoping (*V*_WE_ = 0 mV to −500 mV versus Ag/AgCl) and (**E**) doping (*V*_WE_ = −500 to 0 mV versus Ag/AgCl) showing qualitative agreement with experimental data. Voltage dependence for the |∆*p*/*p*_0_| = 0.5 contour line from the (**F**) classical drift-diffusion model during dedoping and the quasi-field drift-diffusion model during (**G**) dedoping and (**H**) doping.

We simulate dedoping and doping cycles using a one-dimensional electrostatic drift-diffusion model to further capture the experimental results (Fig. 4, A and B). The simulation uses a time-dependent forward finite difference discretization method (*35*) where the electric potential (*V*) at each position (*x*) along the length of the device is computed by solving the Poisson’s equation (Eq. 6) for each time step.

$$\frac{dV^{2}(x)}{{dx}^{2}}=[N_{\mathrm{PSS}}-p(x)-C_{+}(x)]\frac{e}{\varepsilon_{0}\varepsilon_{r}}$$ (6)

where d*V*^2^/d*x*^2^ is the second derivative of the electric potential, *N*_PSS_ is the fixed density of sulfonate sites (2.1 × 10^21^ cm^−3^), *p*(*x*) and *C*_+_(*x*) are the local hole and cation concentrations, respectively, *e* is the elementary charge, and ɛ_0_ and ɛ*_r_* are the vacuum and relative permittivities, respectively. We include a finite length of electrolyte, which is one fourth of the length of the PEDOT:PSS channel, and the concentration of mobile cations and anions correspond to the 0.1 M NaCl solution used experimentally (6.0 × 10^19^ cm^−3^). We use constant *V* condition at the simulation boundaries (*V*_el_ = 0 V, *V*_ITO_ = *V*_WE_) and keep the electrolyte concentration fixed at the simulation boundary. The computed *V*(*x*) is then used to compute the flux of cations and holes. The typical equations used for flux in drift-diffusion models are given in Eqs. 7 and 8 below.

$$J_{C}(x)=-\mu_{C}C_{+}(x)\frac{dV(x)}{dx}-D_{C}\frac{dC_{+}(x)}{dx}$$ (7)

$$J_{p}(x)=-\mu_{p}p(x)\frac{dV(x)}{dx}-D_{p}\frac{dp(x)}{dx}$$ (8)

where μ*_C_* and μ*_p_* are the cation and hole mobilities, respectively, and *D_C_* and *D_p_* are the cation and hole diffusivities, respectively. Often, the diffusion constant is estimated as the product of electrical mobility and thermal voltage (25.9 mV) using the Einstein’s relation (Eq. 9).

$$D\approx \mu \frac{k_{B}T}{q}=\mu \cdot 25.9\;\mathrm{mV}$$ (9)

The resulting drift-diffusion simulation of dedoping using Eq. 9 to estimate diffusivity of holes does not resemble the experimental data (Fig. 4C). The inconsistency between the classical drift-diffusion simulation and experimental data arises from an oversimplification of hole diffusion in a mixed conducting system. Specifically, the Einstein relation (Eq. 8) used to describe hole diffusivity does not capture the thermodynamic energy landscape for holes, which gives the capacitive behavior in the bulk of the material. As discussed earlier in Eq. 4, increasing the concentration of holes results in a shift in the hole quasi-Fermi level ϕ_p_ (*19*). To account for ϕ_p_, we must replace the diffusion term in Eq. 8 to explicitly include the chemical potential gradient, which will generate a quasi-electric field that drives diffusion of holes against the electric field (Fig. 3E). Thus, the diffusion current of holes can be calculated according to Eq. 10.

$$J_{p,\mathrm{diff}}=-\mu_{p}p(x)\frac{d\varphi_{p}(x)}{dx}=-\mu_{p}p(x)\frac{e}{C^{*}}\frac{dp(x)}{dx}$$ (10)

The inclusion of ϕ*_p_* in the diffusion gradient is equivalent to using the general form of the Einstein relation, which states that the diffusion constant of charged particles will depend on the dispersion relationship (e.g., changes in quasi-Fermi level with hole concentration) as described in Eqs. 11 and 12.

$$D_{p}=\mu_{p}p(x)\frac{d\varphi_{p}}{dp}$$ (11)

$$D_{p}=\mu_{p}p(x)\frac{e}{C^{*}}$$ (12)

To ensure *p*(*x*) is always positive, we implement a Gaussian distribution of hole states at tail of highest occupied molecular orbital (HOMO) (*36*) with logarithmically increasing chemical potential as *p*(*x*) approaches 0 (Eq. 13).

$$\varphi_{p}(p)=\varphi_{p}(p_{0})+k_{B}T^{*}\ln\left(\frac{p}{p_{0}}\right)$$ (13)

where we use *p*_0_ = 2 × 10^19^ cm^−3^ as the crossover hole concentration where the simulation switches from computing chemical potential using *C*^∗^ (Eq. 4) to using the Gaussian distribution of states (Eq. 13).** At these lower hole concentrations, ϕ*_p_* is dominated by the limited availability of electronic states at the Gaussian tail of the HOMO band (*36*), where ϕ*_p_* is logarithmically dependent on *p*. We also empirically account for the voltage dependence of μ*_p_* using AC measurements of OECTs on the same substrate as the moving front devices (fig. S2). However, the model still qualitatively matches the dedoping behavior when a constant μ*_p_* value is used (fig. S3).

Last, the simulation must compute hole and ion flux across the boundaries at each end of the film. At OMIEC-electrolyte boundary, the flux of holes and anions is set to 0 and the flux of cations is given by a modified version of Eq. 6, which adds the chemical potential gradient for ions across the interface due to the difference in chemical potential of ions in the OMIEC and electrolyte phases, respectively. This difference in chemical potential is estimated to be 0.3 eV, which gives a built-in potential across the PEDOT:PSS/electrolyte interface of ca. 0.22 V, matching the experimentally measured built in potential. The flux at the boundary between the OMIEC and contact is set to 0 for cations. For holes, the flux into the ITO contact is computed using the difference in electrochemical potential of holes (${\bar{\mu }}_{h}$) across the boundary (Fig. 4B) as described in Eqs. 14 to 16.

$$J_{p,\mathrm{ITO}}=-\mu_{p}p(x=L)\;\frac{{\bar{\mu }}_{p,n}-{\bar{\mu }}_{p,\mathrm{ITO}}}{dx}$$ (14)

$${\bar{\mu }}_{p,n}=eV(x=L)+p(x=L)\frac{e}{C^{*}}$$ (15)

$${\bar{\mu }}_{p,\mathrm{ITO}}=eV_{\mathrm{WE}}$$ (16)

where ${\bar{\mu }}_{p,n}$ and ${\bar{\mu }}_{p,\mathrm{ITO}}$ are the hole electrochemical potential at the polymer and electrode side of the polymer/electrode interface described by Eqs. 15 and 16, respectively.

### Simulation results

The drift-diffusion simulation explicitly accounting for hole quasi-electric fields due to gradients in ϕ*_p_* (which we call the “quasi-field model”) yields dedoping profiles that agree qualitatively with the experimental results (Fig. 4D). We note that the time and length scale for our simulations are much shorter due to the high computational cost. To simulate doping, we use the final state of the dedoping simulation as the initial potential and carrier distribution then set *V*_WE_ to 0 V (Fig. 4E). The doping simulation gives good agreement to the experiment and shows similar asymmetry behavior to experimentally measured doping (Fig. 1E). In contrast, flipping the potential using the classical drift-diffusion model results in limited oxidation as the only driving force is the difference in *C*_+_ between the OMIEC and electrolyte (fig. S4) since there is no potential difference between the electrode, OMIEC, and electrolyte when *V*_WE_ is set to 0 V.

We use the |∆*p*/*p*_0_| = 0.5 contour lines to compare the voltage-dependent (de)doping kinetics predicted by the simulation to the experiment. The contour lines calculated with the classical drift-diffusion model show a strong dependence of the dedoping propagation speed (Fig. 4F). In contrast, the quasi-field model accurately reproduces the lack of voltage dependence for potentials from −0.3 to −0.6 V versus Ag/AgCl and the decrease in dedoping time for higher applied potentials (Fig. 3, G and H).

To leverage the model to infer the ion transport properties for the moving front experiments, we investigate the scaling of the (de)doping kinetics as we scale the channel length, *L*, ion mobility, μ*_C_*, and hole mobility, μ*_p_*. We find that the time scale of dedoping, *t*, scales approximately with *L*^2^ (fig. S5), which is typical for both diffusion and drift limited processes. We also find that *t* scales inversely with μ*_C_* and is independent of μ*_p_* (fig. S6), indicating that cations are the limiting carrier for the dedoping process. The scaling of the dedoping time from figs. S5 and S6 is summarized by Eq. 17.

$$t\propto \frac{L^{2}}{\mu_{C}}$$ (17)

Using Eq. 17, we can extrapolate μ*_C_* for the experimental measurements. If we extrapolate from the *L*-dependent simulations in fig. S5 (μ_C_ = 10^−3^ cm^2^ V^−1^ s^−1^), we predict a dedoping time of ca. 140 ms, which is ca. 40× faster than the experimentally measured dedoping time of ca. 5.5 s. Thus, we can estimate a μ*_C_* of ca. 2.5 × 10^−5^ cm^2^ V^−1^ s^−1^ from our experimental measurements, where μ*_C_* corresponds to the cation mobility in the plane of the film. We expect that this μ*_C_* differs from the out-of-plane ion mobility due to the anisotropy of PEDOT:PSS films (*37*). Last, we note that our simulated scaling is only performed in the range where μ*_p_* >> μ*_C_*, thus we expect that Eq. 17 and the qualitative shape of the (de)doping curves will breakdown as μ*_C_* *C*_+_ approaches μ*_p_* (*27*).

**Fig. 5.**
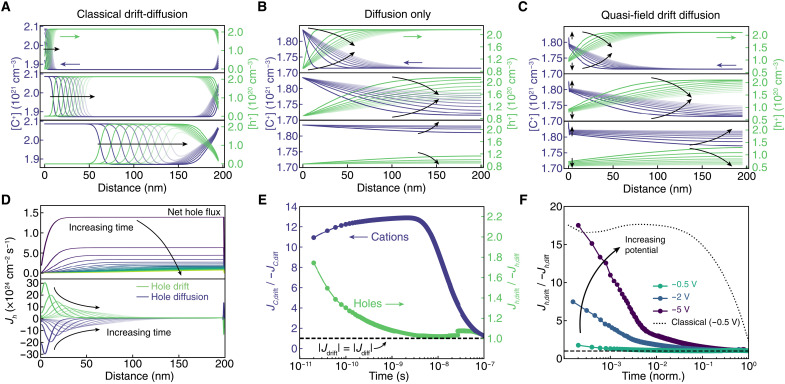
Contributions of drift and diffusion fluxes during dedoping simulations. Cation (purple) and hole (green) concentrations along the length of the polymer channel for the (**A**) classical drift-diffusion model, (**B**) drift-only model, and (**C**) quasi-field drift-diffusion model during simulated dedoping (*V*_WE_ from 0 to −500 mV versus Ag/AgCl). (**D**) Net hole flux, *J_p_*, along the length of the polymer with increasing time (top) and hole flux contributions due to drift (green) and diffusion (purple) with increasing time (bottom). (**E**) The ratio of drift and diffusion fluxes for holes (green) and cations (purple). For holes, drift, *J*_*p*,drift_, dominates at very early time scales, then at longer time scales, diffusion fluxes (*J*_*p*,diff_) fluxes and *J*_*p*,drift_ are nearly equal. In contrast, cation transport is primarily due to drift, *J*_*C*,drift_, over the course of dedoping. (**F**) The ratio of drift and diffusion fluxes for holes increases with increasing driving potential causing dedoping to become dominated by drift. For comparison, the drift ratio is plotted for the classical drift diffusion model (black dotted line). The time scale in (F) is normalized to the dedoping time for each potential (100, 13, and 5 ns, and 20 ns for *V*_WE_ of −0.5, −2, and −5, and −0.5 V classical, respectively). The dashed lines in (E) and (F) denote where drift and diffusion currents are equal (*J*_drit_/−*J*_diff_ = 1).

We compare the dedoping kinetics of the explored models to understand the relative contributions of drift and diffusion fluxes during electrochemical dedoping. If we compare time-dependent hole and ion concentrations computed using the classical drift-diffusion model (Fig. 5A), diffusion-only model (Fig. 5B), and quasi-field drift-diffusion model (Fig. 5C), we find a much closer qualitative agreement between the diffusion and quasi-field drift-diffusion models. If we look at the relative contributions of hole drift and diffusion to the net flux (Fig. 5D), we find that the two fluxes are nearly equal and oppose one another. The positive hole drift is slightly larger than the diffusion, leading to a net flux of holes toward the electrode. Both the drift and diffusion flux profiles along the length of the film broaden with increasing time due to the broadened electric and quasi-electric fields, giving rise to the diffusion-like dedoping behavior.

To identify the dominant driving forces for hole and ion flux, we compare the relative fluxes due to drift and diffusion for our simulations by taking the ratio of the average *J*_drift_ and *J*_diff_ along the entire length of the film (*J*_drift_/−*J*_diff_). For holes (Fig. 5E), drift currents are larger at short time scales, but with increasing time, the relative contribution of diffusion increases until the two fluxes are nearly equal. In contrast to holes, the cation flux is primarily driven by drift during the entire dedoping process. This result can be explained as a leading-following carrier relationship between holes and ions. Since holes have much higher mobilities, they can rearrange under the electric and quasi-electric fields relatively rapidly. Then, net hole flux leads to a local charge imbalance locally, thereby producing an electric field. Last, this electric field drives ion transport, resulting in ion fluxes that are much larger than diffusion of ions alone.

The diffusive dedoping kinetics observed for experiments using device-relevant driving potentials ranging from −0.3 to −0.8 V versus Ag/AgCl contradict previous moving front measurements of ion motion, which appear to be dominated by ion drift (*15*, *20*, *26*, *30*). However, these previous experiments were measured under high driving potentials (−2 V versus Ag/AgCl). To verify that our model can capture this behavior, we simulate dedoping at similarly high driving potentials (*V*_WE_ = −1, −2, and −5 V versus Ag/AgCl). We find that the ratio of drift to diffusion flux increases markedly when a higher potential is used to drive dedoping (Fig. 5F), and the resulting dedoping kinetics appear much closer to the classical drift diffusion simulation (fig. S7). We therefore attribute the discrepancy between our observations and previous experiments to the magnitude of voltage used to drive (de)doping. Use of high potentials leads to drift-like kinetics due to the lack of available electronic states to fill at higher energies, thereby driving electrochemical dedoping with much higher potentials than the equilibrium potential. This result further demonstrates the generality of our quasi-field drift-diffusion model for fitting (de)doping for a wide range of experimental conditions.

## DISCUSSION

We find that hole quasi-electric fields are a substantial driving force dictating the kinetics of electrochemical (de)doping in PEDOT:PSS at typical device operating potentials (ca. ±1 V versus Ag/AgCl). On the basis of this finding, we propose a quasi-field drift-diffusion model that accurately captures the (de)doping kinetics of thin films at device-relevant potentials. At short time scales, the applied potential drives the formation of a depletion region at the interface between PEDOT:PSS and the electrolyte due to the much higher mobility of holes (ca. 0.08 to 1 cm^2^ V^−1^ s^−1^; fig. S2) compared to ions [ca. 1 × 10^−3^ cm^2^ V^−1^ s^−1^ (*20*) to 2.5 × 10^−5^ cm^2^ V^−1^ s^−1^ as predicted in this work]. Initially, this hole motion is primarily drift under the electric field, but the gradients quickly become diffuse due to the high chemical potential associated with sharp hole concentration gradients. Diffusion then broadens the electric field as the quasi-Fermi level of holes in PEDOT:PSS shifts when the carrier concentration is modulated by ion intercalation. Thus, at longer time scales, hole diffusion plays a much larger role in (de)doping than expected from the ideal gas approximation. In contrast to holes, we find that the transport of ions is driven primarily by electrostatics where hole fluxes “lead” and ion fluxes “follow” as hole displacement currents generate an electric field. For this reason, (de)doping occurs much faster in PEDOT:PSS than would be expected for a process limited by only by ion diffusion.

Our quasi-field model predicts that (de)doping speeds only depend on the mobility of the limiting carrier, which is ionic in the case of PEDOT:PSS. However, speeds in devices could potentially be improved by operating in the drift-like regime (fig. S7). For example, a large initial applied bias could be used to inject/extract ions via drift followed by a holding at smaller bias to let the channel system to reach steady state. From a materials perspective, OMIEC design might benefit from higher degree of electronic order, which would give a narrow distribution in the DOS, leading to a higher volumetric capacitance *C*^∗^ (*19*). Since the electronic chemical potential is inversely proportional to *C*^∗^, materials with a narrow DOS could be doped to high charge carrier densities without building up large electronic chemical potential gradients, which push the kinetics to the diffusive regime.

Our experimental results show that electrochemical (de)doping of OMIECs results in the formation of broad depletion/accumulation regions, which are much longer (>100 μm) than predicted for purely electronic systems. This extended space-charge region could be responsible for nonidealities in OECTs, including the nonmonatomic relationship between transconductance (*g*_m_) and gate potential (*38*) and shifts in the gate potential corresponding to the peak in *g*_m_ with changing geometry (*39*). While many nonideal device behaviors may be a result of the dynamic relationship among holes, ions, the electric field, and hole chemical potential, the presented model describing transport in OMIECs justifies the use of capacitive models (*10*), which can accurately predict most of the phenomenological behaviors of OECTs, albeit with unrealistic steady-state conditions (*12*). However, we expect diffusion currents along the channel due to the gradient in the hole quasi-Fermi level to account for a large fraction of the current in OECT channels, indicating that measurement methods for estimating hole mobilities (*40*) may need to be revisited.

There are many improvements that can be made to extend the presented model to understand the structure-property relationships in OMIECs. For example, the model does not explain the more rapid speed of doping compared to dedoping commonly observed optically (*28*, *41*–*43*), which may be related to an intrinsic asymmetry in the transport or thermodynamic coefficients of either holes or ions. Furthermore, the energetic contributions that determine *C^∗^* are likely to be complex, where the DOS (*44*), mixing enthalpy (*45*) and entropy (*46*), strain (*47*), and reorganization energies (*48*) likely all play a role in determining *C^∗^* as a function of voltage. Further work is needed to fully capture all of the complexity of (de)doping in OMIECs, including measurements of strains from ion intercalation (*49*), contact resistance effects (*50*), and composite response in separated phases (*51*). The current model treats the OMIEC as a uniform material with effective transport coefficients, which works well for lengths much larger than the microstructural features, but at short length scales, we expect the model to fail to capture the anisotropy caused by microstructural heterogeneity. Last, extending the drift-diffusion simulations to two dimensions (*12*) with different transport coefficients for the and in- and out-of-plane directions would allow for modeling the more complex behavior of devices such as OECTs.

We expect quasi-electric fields will develop due to ion motion in other mixed ionic-electronic conducting polymers such as intrinsically insulating enhancement-mode OMIECs and inorganic mixed conducting materials. For example, there have been reports of shifts in open-circuit voltage due to shifts in the quasi-Fermi level due to increasing exciton populations in both organic (*52*) and halide perovskite (*53*) solar cells photovoltaic cells. This effect is likely exacerbated in ion conducting systems such as halide perovskites where ion migration can compensate electronic carriers, screening the effect of electrostatic driving forces leading to similar diffusive electronic motion as reported here. Thus, the framework that relates the quasi-Fermi level shifts to the effective driving forces of individual carriers, as well as the leader-follower relationship during electrochemical reactions, will aid in both predicting and understanding the device response more generally in mixed ionic-electronic conductors.

## MATERIALS AND METHODS

### Preparation of PEDOT:PSS

The PEDOT:PSS dispersion was prepared by mixing Clevios PH1000 (Heraeus) with 6% (v/v) ethylene glycol and 1% (v/v) (3-glycidyloxypropyl)trimethoxysilane, sonicating for 10 min, and then filtering through a 0.45-μm polyvinylidene fluoride syringe filter.

### Device fabrication

Fabrication of transparent samples for optical microscopy started with 10 min of sonication of ITO-coated boro-aluminosilicate glass wafers (University Wafer 2544) submerged in acetone followed by isopropyl alcohol (IPA). The contact layer was patterned by coating wafers with AZ 4533 positive photoresist (MicroChemicals) (spin coated at 4000 RPM for 45 s, acceleration of 500 RPM s^−1^, soft bake at 100°C for 50 s) followed by ultraviolet (UV) exposure (198 mJ cm^−2^, Karl Suss MA6 Mask Aligner) and development in AZ 726 MIF developer (MicroChemicals) for 100 s. The pattern was baked at 115°C for 50 s to improve adhesion between the resist and substrate. ITO was etched using a dilute aqua regia ITO etchant solution (A-Gas) for 1 hour and the remaining resist was stripped by washing the substrate with acetone. Conducting polymers were coated onto the patterned ITO wafers (spin coating at 1000 RPM for 40 s, acceleration of 500 RPM s^−1^). The resulting film thicknesses were 224 ± 10 nm. Coated wafers were baked at 120°C for 20 min for cross-linking of (3-glycidyloxypropyl)trimethoxysilane (GOPS) and soaked in deionized water overnight to remove excess PSS molecules. The conducting polymer was patterned into 200-μm-wide, 522.5-μm-long channels (10-μm overlap with ITO contact) using photolithography by coating with AZ 5214E image reversal resist (MicroChemicals) (spin coating at 2500 RPM for 45 s, acceleration of 500 RPM s^−1^, soft bake at 115°C for 120 s) followed by UV exposure (180 mJ cm^−2^) and development in AZ 726 MIF developer for 20 s. The polymer area was defined by reactive ion etching (recipe, Plasma Pro 80 RIE, Oxford Instruments) and the remaining resist was stripped by washing with acetone. The films were exposed to 0.1 M NaCl to allow for passive swelling and uptake of ions. The SU8 capping layer was defined with photolithography by coating the wafers with SU8 2000.5 (Kayaku Advanced Materials, Inc.) (500 RPM for 10 s, acceleration of 100 RPM s^−1^ followed by 2000 RPM for 30 s, acceleration of 300 RPM s^−1^, and then soft baking at 95°C for 60 s) followed by UV exposure (60 mJ cm^−2^) and postexposure bake at 95°C for 90 s. The pattern was developed in propylene glycol monomethyl ether acetate (PGMEA) for 75 s then rinsed with PGMEA then IPA followed by a hard bake at 120°C for 20 min to anneal the SU8 layer. Finished wafers were diced with a diamond scribe and tile cutter tool and a silicone well was defined using an adhesive backed silicone (McMaster-Carr) to confine the electrolyte.

### Optical transmission microscopy

Optical microscopy was performed with a home-built inverted widefield transmission microscope. The sample was illuminated from the top using a broadband light source (Fiber-Lite DC-950) focused onto the sample with an OSL2 fiber bundle focusing package (ThorLabs OSL2FOC). The sample was fixed to a piezo-driven sample stage (Attocube ECSx5050/AL/RT/NUM) to control the position of the sample in the *x*, *y*, and *z* axes and the transmitted light was captured with a 10× objective (Olympus PLN 10× objective, 0.25 numerical aperture) that was fixed to the optical table. For grayscale imaging, light was imaged by a tube lens [300-mm focal length (FL), VIS-NIR–coated achromat, Edmund Optics] onto the camera (FLIR, Grasshopper3, GS3-U3-23S6M-C). The magnification was 16.7× (350 nm per pixel). The image acquisition was controlled using custom LabView code.

For spectral resolution, the imaging system was extended by a 1:1 telecentric lens pair (300-mm FL, VIS-NIR–coated achromat, Edmund Optics) to gain access to an intermediate image and back focal plane (*54*). The transmitted light first passed through a diffraction-limited slit placed in the intermediate imaging plane. The slit was aligned along the long axis of the sample. Afterward, an F2 prism (PS852, Thorlabs) placed in the intermediate back focal plane dispersed the transmitted light orthogonally to the slit before being imaged onto a 16-bit scientific complementary metal-oxide semiconductor camera (Hamamatsu, ORCA Flash 4 V3). The overall magnification was 16.7× (390 nm per pixel) and spectral calibration was achieved through a series of bandpass filters (FBXXX-10, Thorlabs) across the visible spectral region. The image acquisition was controlled using custom LabView code.

### Electrochemistry

Chronoamperometry was performed using a Gamry Instruments Interface 1010E Potentiostat. The sample was connected to the working electrode and a cylindrical Ag/AgCl pellet electrode (2 mm diameter, 4 mm length) was used as the combined reference and counter electrode. The initial voltage (*V*_WE,*i*_) was held for 5 min before applying the final voltage (*V*_WE,*f*_,) at which the moving front measurement was performed. All experiments were performed using 0.1 M NaCl dissolved in water as the electrolyte.

### Electrical characterization

Electrochemical transistors were characterized using a Keysight B2902A Source-Measure Unit controlled custom Python code. Mobility and capacitance were measured using OECTs on the same wafer using a previously reported frequency dependent measurement (*40*). All experiments were performed using 0.1 M NaCl dissolved in water as the electrolyte.

### Film thickness measurements

Film thicknesses were measured using a DekTak XT Profilometer with a scan rate of 17 μm s^−1^ and a stylus force of 1 mg. Each sample was measured in six locations and averaged.

### Simulation procedures

Drift diffusion simulations (*35*) were carried out as described in the Supplementary Text using custom Python code. Physical parameters used in the simulations are summarized in table S2.

## Notes

**Correction (3 November 2023):** The original version of Eq. 11 used elementary charge (*e*) as a correction factor. This has been corrected to be consistent with other equations in the paper. The PDF and HTML have been updated.

## Supplementary Materials

### This PDF file includes:

Supplementary Text Figs. S1 to S7 Tables S1 and S2 References

## References

[1] A. Sood, A. D. Poletayev, D. A. Cogswell, P. M. Csernica, J. T. Mefford, D. Fraggedakis, M. F. Toney, A. M. Lindenberg, M. Z. Bazant, W. C. Chueh, Electrochemical ion insertion from the atomic to the device scale. Nat. Rev. Mater. 6, 847–867 (2021).

[2] S. T. Keene, V. Gueskine, M. Berggren, G. G. Malliaras, K. Tybrandt, I. Zozoulenko, Exploiting mixed conducting polymers in organic and bioelectronic devices. Phys. Chem. Chem. Phys. 24, 19144–19163 (2022). 35942679 10.1039/d2cp02595g

[3] S. L. Bidinger, S. T. Keene, S. Han, K. W. Plaxco, G. G. Malliaras, T. Hasan, Pulsed transistor operation enables miniaturization of electrochemical aptamer-based sensors. Sci. Adv. 8, eadd4111 (2022). 36383656 10.1126/sciadv.add4111 PMC9668304

[4] S. T. Keene, D. Fogarty, R. Cooke, C. D. Casadevall, A. Salleo, O. Parlak, Wearable organic electrochemical transistor patch for multiplexed sensing of calcium and ammonium ions from human perspiration. Adv. Healthc. Mater. 8, 1901321 (2019). 10.1002/adhm.201901321 31714014

[5] Y. van de Burgt, E. Lubberman, E. J. Fuller, S. T. Keene, G. C. Faria, S. Agarwal, M. J. Marinella, A. Alec Talin, A. Salleo, A non-volatile organic electrochemical device as a low-voltage artificial synapse for neuromorphic computing. Nat. Mater. 16, 414–418 (2017). 28218920 10.1038/nmat4856

[6] A. A. Talin, Y. Li, D. A. Robinson, E. J. Fuller, S. Kumar, ECRAM materials, devices, circuits and architectures: A perspective. Adv. Mater., 2204771 (2022). 10.1002/adma.202204771 36354177

[7] D. Moia, A. Giovannitti, A. A. Szumska, I. P. Maria, E. Rezasoltani, M. Sachs, M. Schnurr, P. R. F. Barnes, I. McCulloch, J. Nelson, Design and evaluation of conjugated polymers with polar side chains as electrode materials for electrochemical energy storage in aqueous electrolytes. Energy Environ. Sci. 12, 1349–1357 (2019).

[8] C. M. Proctor, J. Rivnay, G. G. Malliaras, Understanding volumetric capacitance in conducting polymers. J. Polym. Sci. B 54, 1433–1436 (2016).

[9] A. V. Volkov, K. Wijeratne, E. Mitraka, U. Ail, D. Zhao, K. Tybrandt, J. W. Andreasen, M. Berggren, X. Crispin, I. V. Zozoulenko, Understanding the capacitance of PEDOT:PSS. Adv. Funct. Mater. 27, 1700329 (2017).

[10] D. A. Bernards, G. G. Malliaras, Steady-state and transient behavior of organic electrochemical transistors. Adv. Funct. Mater. 17, 3538–3544 (2007).

[11] J. T. Friedlein, S. E. Shaheen, G. G. Malliaras, R. R. McLeod, Optical measurements revealing nonuniform hole mobility in organic electrochemical transistors. Adv. Electron. Mater. 1, 1500189 (2015).

[12] V. Kaphle, P. R. Paudel, D. Dahal, R. K. Radha Krishnan, B. Lüssem, Finding the equilibrium of organic electrochemical transistors. Nat. Commun. 11, 2515 (2020). 32433542 10.1038/s41467-020-16252-2 PMC7239912

[13] K. Tybrandt, I. V. Zozoulenko, M. Berggren, Chemical potential–electric double layer coupling in conjugated polymer–polyelectrolyte blends. Sci. Adv. 3, eaao3659 (2017). 29260000 10.1126/sciadv.aao3659 PMC5734606

[14] B. D. Paulsen, K. Tybrandt, E. Stavrinidou, J. Rivnay, Organic mixed ionic–electronic conductors. Nat. Mater. 19, 13–26 (2020). 31427743 10.1038/s41563-019-0435-z

[15] J. Rivnay, S. Inal, B. A. Collins, M. Sessolo, E. Stavrinidou, X. Strakosas, C. Tassone, D. M. Delongchamp, G. G. Malliaras, Structural control of mixed ionic and electronic transport in conducting polymers. Nat. Commun. 7, 11287 (2016). 27090156 10.1038/ncomms11287 PMC4838877

[16] S. T. Keene, W. Michaels, A. Melianas, T. J. Quill, E. J. Fuller, A. Giovannitti, I. McCulloch, A. A. Talin, C. J. Tassone, J. Qin, A. Troisi, A. Salleo, Efficient electronic tunneling governs transport in conducting polymer-insulator blends. J. Am. Chem. Soc. 144, 10368–10376 (2022). 35658455 10.1021/jacs.2c02139 PMC9204759

[17] D. Tu, S. Fabiano, Mixed ion-electron transport in organic electrochemical transistors. Appl. Phys. Lett. 117, 080501 (2020).

[18] H. Kroemer, Quasi-electric fields and band offsets: Teaching electrons new tricks (Nobel Lecture). ChemPhysChem 2, 490–499 (2001). 23686986 10.1002/1439-7641(20010917)2:8/9<490::AID-CPHC490>3.0.CO;2-1

[19] S. Yu, E. L. Ratcliff, Tuning organic electrochemical transistor (OECT) transconductance toward zero gate voltage in the faradaic mode. ACS Appl. Mater. Interfaces 13, 50176–50186 (2021). 34644052 10.1021/acsami.1c13009

[20] E. Stavrinidou, P. Leleux, H. Rajaona, D. Khodagholy, J. Rivnay, M. Lindau, S. Sanaur, G. G. Malliaras, Direct measurement of ion mobility in a conducting polymer. Adv. Mater. 25, 4488–4493 (2013). 23784809 10.1002/adma.201301240

[21] S. T. Keene, A. Melianas, E. J. Fuller, Y. van de Burgt, A. A. Talin, A. Salleo, Optimized pulsed write schemes improve linearity and write speed for low-power organic neuromorphic devices. J. Phys. D Appl. Phys. 51, 224002 (2018).

[22] E. J. Fuller, S. T. Keene, A. Melianas, Z. Wang, S. Agarwal, Y. Li, Y. Tuchman, C. D. James, M. J. Marinella, J. J. Yang, A. Salleo, A. A. Talin, Parallel programming of an ionic floating-gate memory array for scalable neuromorphic computing. Science 364, 570–574 (2019). 31023890 10.1126/science.aaw5581

[23] J. D. Slinker, J. A. DeFranco, M. J. Jaquith, W. R. Silveira, Y. W. Zhong, J. M. Moran-Mirabal, H. G. Craighead, H. D. Abruña, J. A. Marohn, G. G. Malliaras, Direct measurement of the electric-field distribution in a light-emitting electrochemical cell. Nat. Mater. 6, 894–899 (2007). 17906631 10.1038/nmat2021

[24] J. C. Demello, N. Tessler, S. C. Graham, R. H. Friend, Ionic space-charge effects in polymer light-emitting diodes. Phys. Rev. B 57, 12951–12963 (1998).

[25] S. van Reenen, P. Matyba, A. Dzwilewski, R. A. J. Janssen, L. Edman, M. Kemerink, A unifying model for the operation of light-emitting electrochemical cells. J. Am. Chem. Soc. 132, 13776–13781 (2010). 20831189 10.1021/ja1045555

[26] S. Inal, G. G. Malliaras, J. Rivnay, Optical study of electrochromic moving fronts for the investigation of ion transport in conducting polymers. J. Mater. Chem. C 4, 3942–3947 (2016).

[27] S. T. Keene, J. E. M. Laulainen, R. Pandya, M. Moser, C. Schnedermann, P. A. Midgley, I. McCulloch, A. Rao, G. G. Malliaras, Hole-limited electrochemical doping in conjugated polymers. Nat. Mater. 10.1038/s41563-023-01601-5 (2023). PMC10465356 37414944

[28] G. Rebetez, O. Bardagot, J. Affolter, J. Réhault, N. Banerji, What drives the kinetics and doping level in the electrochemical reactions of PEDOT:PSS? Adv. Funct. Mater. 32, 2105821 (2022).

[29] R. Wu, B. D. Paulsen, Q. Ma, J. Rivnay, Mass and charge transport kinetics in an organic mixed ionic–electronic conductor. Chem. Mater. 34, 9699–9710 (2022).

[30] E. Stavrinidou, P. Leleux, H. Rajaona, M. Fiocchi, S. Sanaur, G. G. Malliaras, A simple model for ion injection and transport in conducting polymers. J. Appl. Phys. 113, 244501 (2013).

[31] C. G. Bischak, L. Q. Flagg, D. S. Ginger, Ion exchange gels allow organic electrochemical transistor operation with hydrophobic polymers in aqueous solution. Adv. Mater. 32, 2002610 (2020). 10.1002/adma.202002610 32596942

[32] S. Han, S. Yamamoto, A. G. Polyravas, G. G. Malliaras, Microfabricated ion-selective transistors with fast and super-nernstian response. Adv. Mater. 32, 2004790 (2020). 10.1002/adma.202004790 33118196

[33] Y. Zhang, M. Nguyen, C. Schnedermann, S. T. Keene, I. Jacobs, A. Rao, H. Sirringhaus, Transmission-based charge modulation microscopy on conjugated polymer blend field-effect transistors. J. Chem. Phys. 158, 034201 (2023). 36681638 10.1063/5.0132426

[34] A. Giovannitti, R. B. Rashid, Q. Thiburce, B. D. Paulsen, C. Cendra, K. Thorley, D. Moia, J. T. Mefford, D. Hanifi, D. Weiyuan, M. Moser, A. Salleo, J. Nelson, I. McCulloch, J. Rivnay, Energetic control of redox-active polymers toward safe organic bioelectronic materials. Adv. Mater. 32, 1908047 (2020). 10.1002/adma.201908047 32125736

[35] C. Bonfil, Carleton University (2014).

[36] H. Bässler, D. Kroh, F. Schauer, V. Nádaždy, A. Köhler, Mapping the density of states distribution of organic semiconductors by employing energy resolved–electrochemical impedance spectroscopy. Adv. Funct. Mater. 31, 2007738 (2021).

[37] A. M. Nardes, M. Kemerink, R. A. J. Janssen, J. A. M. Bastiaansen, N. M. M. Kiggen, B. M. W. Langeveld, A. J. J. M. van Breemen, M. M. de Kok, Microscopic understanding of the anisotropic conductivity of PEDOT:PSS thin films. Adv. Mater. 19, 1196–1200 (2007).

[38] Jacob T. Friedlein, Jonathan Rivnay, David H. Dunlap, Iain McCulloch, Sean E. Shaheen, Robert R. McLeod, George G. Malliaras, Influence of disorder on transfer characteristics of organic electrochemical transistors. Applied Physics Letters 111 2017). 10.1063/1.4993776.

[39] J. Rivnay, P. Leleux, M. Sessolo, D. Khodagholy, T. Hervé, M. Fiocchi, G. G. Malliaras, Organic electrochemical transistors with maximum transconductance at zero gate bias. Adv. Mater. 25, 7010–7014 (2013). 24123258 10.1002/adma.201303080

[40] D. Ohayon, V. Druet, S. Inal, A guide for the characterization of organic electrochemical transistors and channel materials. Chem. Soc. Rev. 52, 1001–1023 (2023). 36637165 10.1039/d2cs00920j

[41] B. D. Paulsen, R. Wu, C. J. Takacs, H. G. Steinrück, J. Strzalka, Q. Zhang, M. F. Toney, J. Rivnay, Time-resolved structural kinetics of an organic mixed ionic–electronic conductor. Adv. Mater. 32, e2003404 (2020). 32864811 10.1002/adma.202003404

[42] V. Jain, H. M. Yochum, R. Montazami, J. R. Heflin, Millisecond switching in solid state electrochromic polymer devices fabricated from ionic self-assembled multilayers. Appl. Phys. Lett. 92, 033304 (2008).

[43] J. Y. Kim, J.-Y. Oh, S. Cheon, H. Lee, J. Lee, J.-I. Lee, H. Ryu, S. M. Cho, T.-Y. Kim, C.-S. Ah, Y.-H. Kim, C.-S. Hwang, Optimized ion diffusion depth for maximizing optical contrast of environmentally friendly PEDOT:PSS electrochromic devices. Opt. Mater. Express 6, 3127–3134 (2016).

[44] I. Sahalianov, S. K. Singh, K. Tybrandt, M. Berggren, I. Zozoulenko, The intrinsic volumetric capacitance of conducting polymers: Pseudo-capacitors or double-layer supercapacitors? RSC Adv. 9, 42498–42508 (2019). 35542835 10.1039/c9ra10250g PMC9076818

[45] P. Shiri, E. J. S. Dacanay, B. Hagen, L. G. Kaake, Vogel–Tammann–Fulcher model for charging dynamics in an organic electrochemical transistor. J. Mater. Chem. C 7, 12935–12941 (2019).

[46] M. Cucchi, A. Weissbach, L. M. Bongartz, R. Kantelberg, H. Tseng, H. Kleemann, K. Leo, Thermodynamics of organic electrochemical transistors. Nat. Commun. 13, 4514 (2022). 35922437 10.1038/s41467-022-32182-7 PMC9349225

[47] X. Wang, X. Li, J. Mei, K. Zhao, Doping kinetics in organic mixed ionic–electronic conductors: Moving front experiments and the stress effect. Extreme Mech. Lett. 54, 101739 (2022).

[48] A. Khot, B. M. Savoie, How side-chain hydrophilicity modulates morphology and charge transport in mixed conducting polymers. J. Polym. Sci. 60, 610–620 (2022).

[49] L. Q. Flagg, L. E. Asselta, N. D’Antona, T. Nicolini, N. Stingelin, J. W. Onorato, C. K. Luscombe, R. Li, L. J. Richter, In situ studies of the swelling by an electrolyte in electrochemical doping of ethylene glycol-substituted polythiophene. ACS Appl. Mater. Interfaces 14, 29052–29060 (2022). 35696277 10.1021/acsami.2c06169

[50] V. Kaphle, S. Liu, A. Al-Shadeedi, C.-M. Keum, B. Lüssem, Contact resistance effects in highly doped organic electrochemical transistors. Adv. Mater. 28, 8766–8770 (2016). 27511804 10.1002/adma.201602125

[51] Y. Cao, G. Yu, A. J. Heeger, C. Y. Yang, Efficient, fast response light-emitting electrochemical cells: Electroluminescent and solid electrolyte polymers with interpenetrating network morphology. Appl. Phys. Lett. 68, 3218–3220 (1996).

[52] D. B. Riley, O. J. Sandberg, N. M. Wilson, W. Li, S. Zeiske, N. Zarrabi, P. Meredith, R. Österbacka, A. Armin, Direct quantification of quasi-fermi-level splitting in organic semiconductor devices. Phys. Rev. Appl. 15, 064035 (2021).

[53] P. Caprioglio, M. Stolterfoht, C. M. Wolff, T. Unold, B. Rech, S. Albrecht, D. Neher, On the relation between the open-circuit voltage and quasi-fermi level splitting in efficient perovskite solar cells. Adv. Energy Mater. 9, 1901631 (2019).

[54] A. Weigel, A. Sebesta, P. Kukura, Dark field microspectroscopy with single molecule fluorescence sensitivity. ACS Photonics 1, 848–856 (2014).

[55] G. LeCroy, C. Cendra, T. J. Quill, M. Moser, R. Hallani, J. F. Ponder, K. Stone, S. D. Kang, A. Y.-L. Liang, Q. Thiburce, I. McCulloch, F. C. Spano, A. Giovannitti, A. Salleo, Role of aggregates and microstructure of mixed-ionic–electronic-conductors on charge transport in electrochemical transistors. Mater. Horiz. 10, 2568–2578 (2023). 37089107 10.1039/d3mh00017f

