# Supplementary material for: The relationship between ionic-electronic coupling and transport in organic mixed conductors
Source: Sci Adv. 2023 Aug 30;9(35):eadi3536. doi: 10.1126/sciadv.adi3536 (PMC10468126; doi:10.1126/sciadv.adi3536)
Supplement: Supplementary file 1 — Supplementary Text Figs. S1 to S7 Tables S1 and S2 References [file sciadv.adi3536_sm.pdf]

Science Advances
AAAS

Supplementary Materials for

The relationship between ionic-electronic coupling and transport in organic mixed conductors

Scott T. Keene *et al.*

Corresponding author: Scott T. Keene, stk30@cam.ac.uk, George G. Malliaras, gm603@cam.ac.uk

Sci. Adv. **9**, eadi3536 (2023)
DOI: 10.1126/sciadv.adi3536

This PDF file includes:

Supplementary Text
Figs. S1 to S7
Tables S1 and S2
References

Supplementary Text

Description of finite difference time-dependent drift-diffusion simulations

To model electrochemical doping and dedoping in PEDOT:PSS, we use a finite difference time-dependent drift-diffusion procedure as previously described (*35*). We note that the time and length scales of our model are much lower than the measured devices due to the large computational cost for simulating longer devices. Our model starts with the determination of the potential profile along the channel length (x) using Poisson's equation (**Eq. S1**) to describe the potential, where all potentials are in reference to the grounded Ag/AgCl working/counter electrode.

$$\frac{d^2V(x)}{dx^2} = (N_{Pss} - p(x) - C_+(x))\frac{e}{\epsilon_0\epsilon_r} \quad (S1)$$

Where $\frac{d^2V(x)}{dx^2}$ is the second derivative of the potential, N_{PSS} is concentration of fixed anionic sites in PEDOT:PSS, p(x) and C_+(x) are the hole and cation concentrations at each length increment dx, e is the elementary charge, and ϵ_r and ϵ_0 are the relative and vacuum permittivities, respectively. We use a constant potential boundary condition (**Eq. S2**) to solve Poisson's equation for each time increment, where the boundaries are in the electrolyte (away from the polymer/electrolyte interface) and at the ITO contact at the interface with PEDOT:PSS. The width of electrolyte modeled is sufficiently wide (50 nm) to have the electric double layer at the polymer-electrolyte interface captured in the simulation.

$$V_{left} = 0\,V\,;\ V_{right} = V_{WE} \quad (S2)$$

We solve Poisson's equation at each time step using LU matrix decomposition method to get the potential along the channel, $V(x)$. Then, the electric field is computed for each box using finite differences method described in **Eq. S3** below.

$$E(x) = \frac{V(x - dx) - V(x + dx)}{2dx} \quad (S3)$$

For simulations incorporating chemical potential gradients, the chemical potential is computed for each distance increment (dx). The chemical potential is referenced to the initial hole concentration (when V_{WE} = 0 V) (p_0) as a reference for φ_p = 0 V.

$$\varphi_p(x) = \frac{p(x) - p_0}{C^*}e \quad (S4)$$

Where C^* is the volumetric capacitance of PEDOT:PSS. To ensure hole concentrations do not go below 0, we model the tail of the density of states as a Gaussian distribution such that the quasi-Fermi level scales with the natural log of the carrier concentration.

$$\varphi_p(p) = \varphi_p(p) + k_BT * ln\left(\frac{p}{p_0}\right) \quad (S5)$$

Once the potential landscape is computed, the hole and ion fluxes computed for each boundary between distance increments along the length of the film using **Eq. S6** and **Eq. S7**.

$$J_p(x) = \mu_p p\left(E - \frac{d\varphi_p}{dx}\right) \quad (S6)$$

$$J_C = \mu_C \left(CE - V_{kBT} \frac{dC}{dx} \right) \qquad (S7)$$

At the boundaries of the film, we use a reflecting boundary for holes at the electrolyte interface and a chemical potential gradient to determine the flux across the electrode interface as described in **Eq. S8**.

$$J_{p,el} = 0\ ;\ J_{p,ITO} = -\mu_p p \left(\frac{V(L) + \varphi_p(L) - V_{ITO}}{dx} \right) \qquad (S8)$$

For ions, we use a reflecting boundary for cations at the electrode interface and a reflecting boundary for anions at the electrolyte interface as described in **Eq. S9**.

$$J_{C,ITO} = 0;\ J_{N,el} = 0 \qquad (S9)$$

At the electrolyte interface, we account for the built in potential between the PEDOT:PSS and electrolyte by using a constant offset in chemical potential of cations within PEDOT:PSS of 0.3 V. This change in chemical potential acts effectively as a quasi-electric field for cations localized at the electrolyte interface. To avoid sharp gradients which make the simulation unstable, the chemical potential of cations is increased over five distance increments rather than as one sharp step.

Once the fluxes are computed, the concentrations in each distance increment are updated using **Eq. S10** and **Eq. S11**.

$$C(x, t + dt) = C(x, t) + \frac{dt}{dx}\left(J_{C,x-1} - J_{C,x}\right) \qquad (S10)$$

$$p(x, t + dt) = p(x, t) + \frac{dt}{dx}\left(J_{p,x-1} - J_{p,x}\right) \qquad (S11)$$

The procedure is then repeated *n* times to get the time-dependent (de)doping behavior, where n is chosen to capture the full kinetics of the doping or dedoping process.

To determine the initial distribution of holes and ions, we run the simulation with V_{WE} = 0 V. For reduction (dedoping) simulations, we use the final distribution of carriers from the simulation at V_{WE} = 0 V and set V_{WE} to the reducing potential (-0.3 V to -0.8 V). For oxidation (doping) simulations, we use the final distribution of holes and ions as the starting point and set the V_{WE} to 0 V.

<u>Interpretation of optical results and linear relationship between transmission and carrier density</u>
Within the spectral region probed (1.5 eV to 2.6 eV), the absorption of the sample is dominated by the neutral PEDOT species. There also appears to be an isosbestic point for the transition between neutral species and polarons at *ca.* 1.55 eV (**Fig. 2a**). Therefore, we attribute the changes in transmitted intensity to changes in the number of neutral PEDOT chains (absorbing species number density, *n*). For a single photon energy (*E*), the transmitted light intensity is given by **Eq. S12**.

$$I(E) = I_i(E) * e^{-\tau} \qquad (S12)$$

Where $I(E)$ is the transmitted intensity, $I_i(E)$ is the incident light intensity, and τ is the attenuation coefficient which, according to the Beer-Lambert law, is given by **Eq. S13**.

$$\tau(E) = \sigma(E)\int_0^l n(z)dz \qquad (S13)$$

Where σ is the attenuation cross section, l is the thickness of the film, and z is the distance along the z-axis through the film. If we assume that the absorbers (neutral PEDOT chains) are uniformly distributed through the thickness of the film, we can simplify τ to **Eq. S14**.

$$\tau(E) = \sigma(E)nl \qquad (S14)$$

If we also assume that, for each wavelength, $\sigma(E)$ and l are constants, we can further simplify the changes in transmitted intensity with **Eq. S15** and **Eq. S16**.

$$\frac{\Delta T}{T_0}(E) = \frac{I_i e^{-\sigma(E)ln_1} - I_i e^{-\sigma(E)ln_0}}{I_i e^{-\sigma(E)ln_0}} = \frac{e^{-\sigma(E)ln_1}}{e^{-\sigma(E)ln_0}} - 1 = e^{-\sigma(E)l(n_1-n_0)} - 1 \qquad (S15)$$

$$\frac{\Delta T}{T_0}(E) = e^{-\sigma(E)l\Delta n} - 1 \qquad (S16)$$

For small values of the exponential function, we can use the following simplification (**Eq. S17**).

$$e^x \approx 1 + x, \qquad \frac{\Delta T}{T_0}(E) \approx -\sigma(E)l\Delta n \qquad (S17)$$

This assumption only deviates by about 3% at the peak in the differential transmission spectrum ($\Delta T/T_0 = 28\%$).

Last, the integrated $\Delta T/T_0$ over the full spectral region can be calculated using the below equation, where we expect the function $\sigma(E)$ to be independent of the number density of neutral PEDOT chains n according to **Eq. S18**.

$$\int_{E_1}^{E_2} \frac{\Delta T}{T_0}(E)dE = l\Delta n \int_{E_1}^{E_2} -\sigma(E)dE\,, \qquad \int_{E_1}^{E_2} -\sigma(E)dE = constant \qquad (S18)$$

Which gives the following linear relationship between the number density of neutral PEDOT absorbers and the relative change in transmitted intensity described in **Eq. S19**.

$$\int_{E_1}^{E_2} \frac{\Delta T}{T_0}(E)dE \propto \Delta n \qquad (S19)$$

Then because we know that changes in carrier density Δp are linearly proportional to applied potential, it is not surprising that the change in neutral PEDOT chains is also proportional to applied potential. So, we expect the linear relationship between $\Delta T/T_0$ and Δp to hold until one of the following assumptions is no longer valid.

1) The linear relationship between $\Delta T/T_0$ and the number density of neutral polymer chromophores is only expected in the spectral region where either (a) no other species absorb, or (b) the absorbing species at the probed photon energies is not changing in number density.

2) All changes in carrier concentration, Δp, have a corresponding inverse change in neutral PEDOT chromophore concentration, Δn. In other words, all doping or dedoping involves conversion between oxidized and neutral PEDOT chains. Thus, as multi-polaron states form at high carrier densities, we do not expect a linear relationship between Δp and $\Delta T/T_0$.

3) The distribution of neutral PEDOT chains is uniform through the thickness of the film. Therefore, this methodology may need to be adjusted for monitoring of ion transport along the optical axis.

4) The attenuation cross-section of the absorbing neutral PEDOT species is constant with changing oxidation state. In other words, oxidation of PEDOT chains only lowers the number density of chromophores but does not interfere with chromophore absorption.

5) The spectral dependence of the attenuation cross-section does not depend on oxidation state. Therefore, the analysis may need to account for spectral shifts in neutral chain absorption line shape with (de)doping which may result from different chromophore populations which dope at different potentials (*55*).

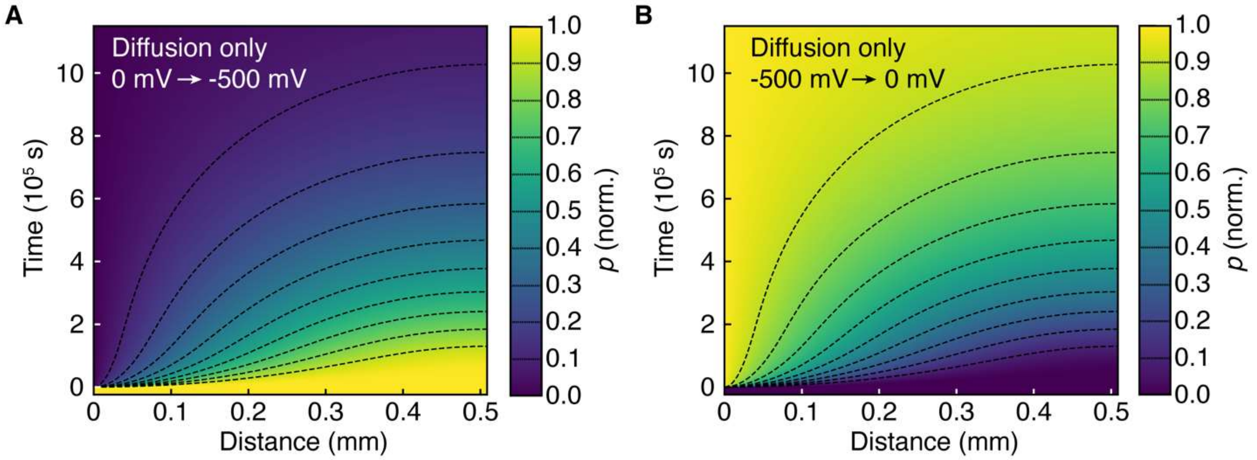

Fig. S1. Simulation of (de)doping of PEDOT:PSS considering only cation diffusion.

Finite difference method for simulation of (**A**) dedoping (V_{WE} from 0 mV to -500 mV *vs.* Ag/AgCl) and (**B**), doping (V_{WE} from -500 mV to 0 mV *vs.* Ag/AgCl) fronts using diffusion only. The model sets the concentration of cations at the interface between PEDOT:PSS and the electrolyte to the ion concentration corresponding to the final voltage applied. The final cation concentration is calculated based on the volumetric capacitance, and the plotted normalized hole concentration (p) is the one minus the normalized cation concentration.

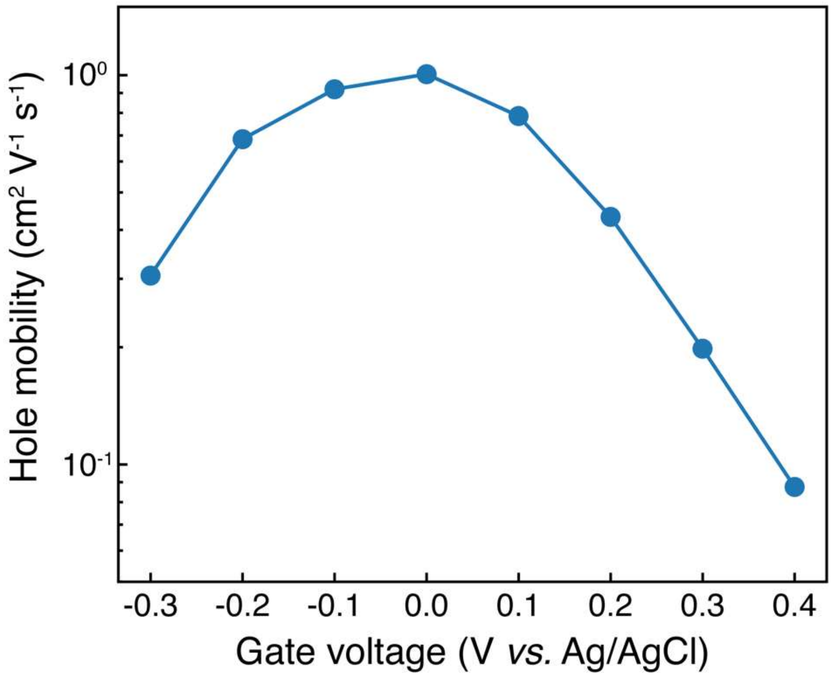

Fig. S2. Voltage-dependent hole mobility in PEDOT:PSS.

Hole mobility measured using an AC gate signal about the offset potential. Note that the polarity of the gate voltage is inverted compared to moving front experiments where the voltage V_{WE} is applied to the PEDOT:PSS film.

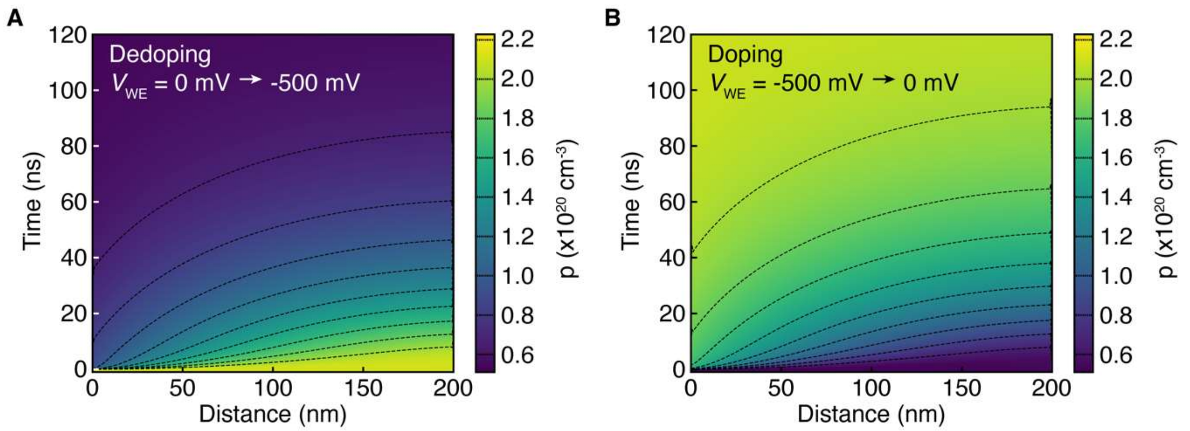

Fig. S3. Quasi-field drift-diffusion simulations with a fixed hole mobility.
Drift-diffusion simulations carried out using the same methodology as those in **Fig. 4** except with a constant value of 1 cm^2 V^{-1} s^{-1} for the hole mobility for (**A**) dedoping and (**B**) doping. When the variable mobility is not accounted for, the model does not capture the asymmetry between dedoping and doping observed experimentally.

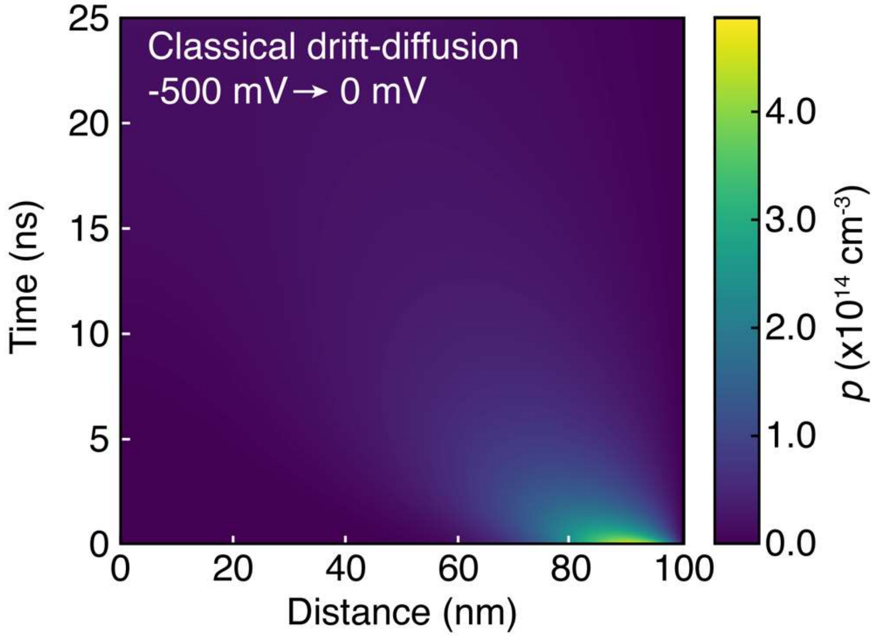

Fig. S4. Classical drift-diffusion simulation of doping.

Doping is simulated using the classical drift-diffusion model starting from the dedoped state. At the initial state, the potential across the entire device is 0 V. Therefore, there is no driving force for doping resulting in very small changes in the hole concentration (< 10^{15} cm^{-3}) over the course of the simulation.

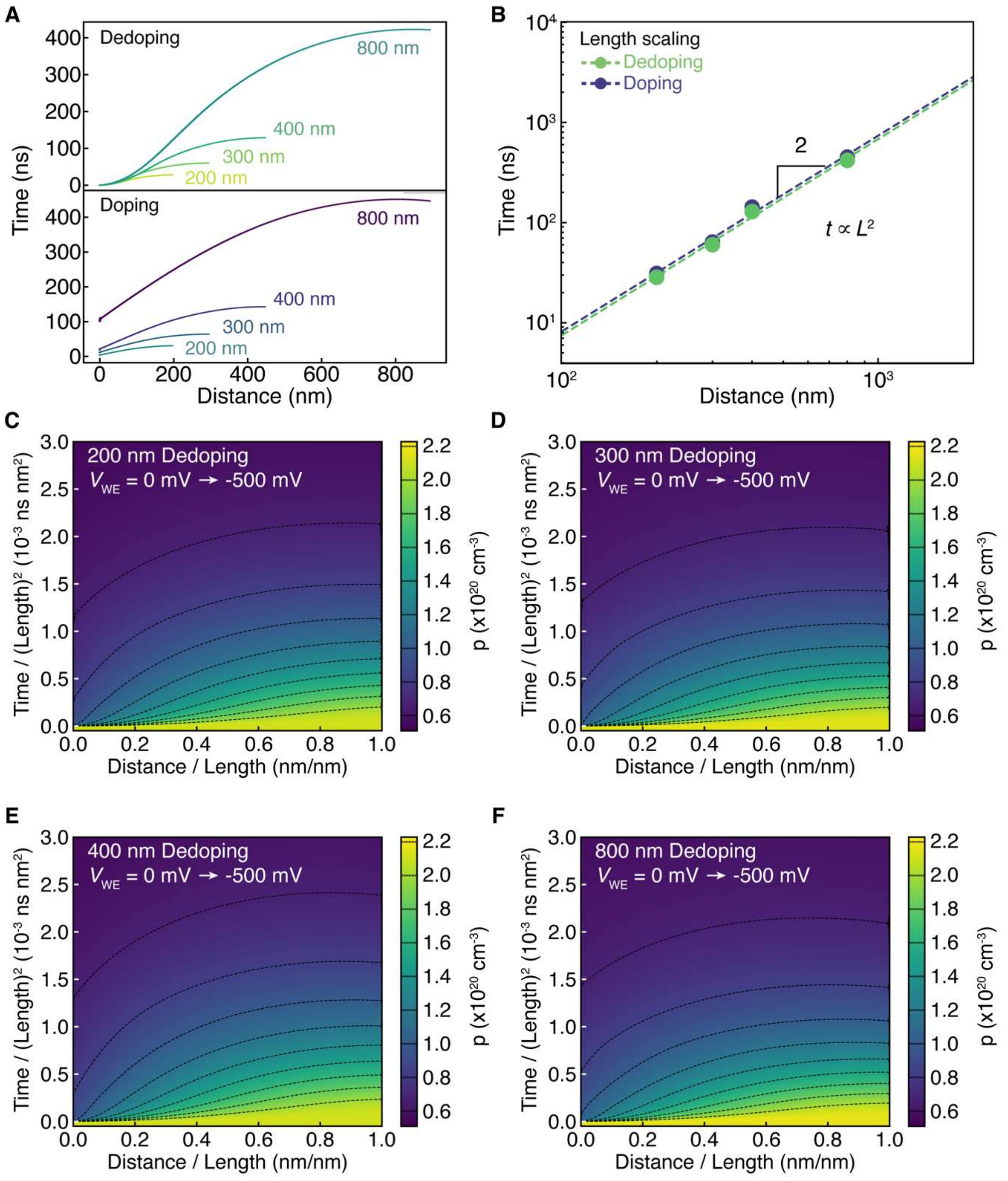

Fig. S5. Quasi-field drift-diffusion simulations with increasing channel length.
(**A**) Quasi-field drift-diffusion simulations for PEDOT:PSS channels with increasing length and (**B**) the corresponding relationship between the dedoping and doping time (t) and channel length (L) showing a relationship of $L^{1.96} \propto t$ which is near the expected length scaling for either a diffusion of drift limited process ($t \propto L^2$). The normalized plots of the quasi-field dedoping simulations for (**C**) L = 200 nm, (**D**) L = 300 nm, (**E**) L = 400 nm, and (**F**) L = 800 nm, also show that the dedoping time, t, scales approximately with L^2.

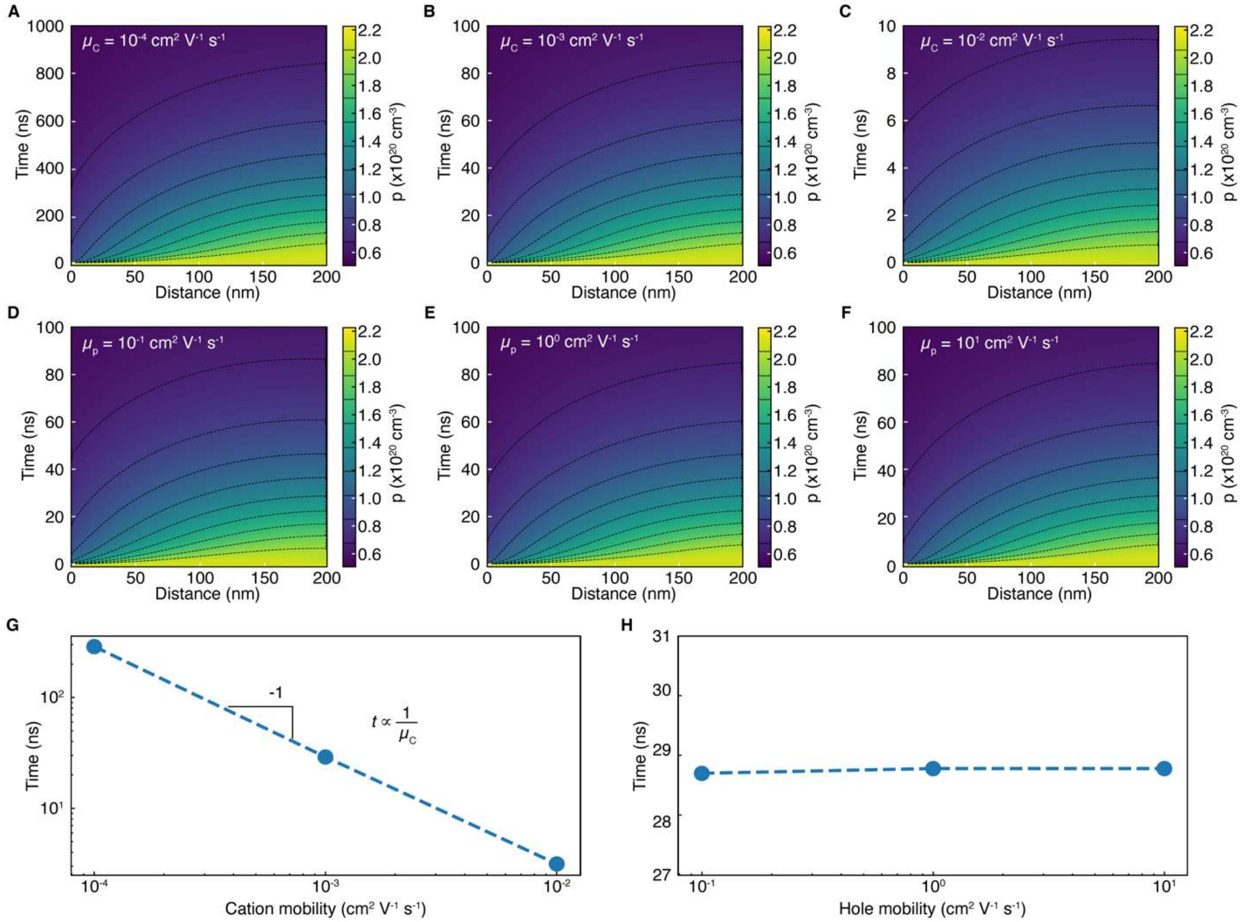

Fig. S6. Quasi-field drift-diffusion simulations with varied cation and hole mobility. Simulations of dedoping with cation mobilities of (**A**) 10^{-4} cm^2 V^{-1} s^{-1}, (**B**) 10^{-3} cm^2 V^{-1} s^{-1}, and (**C**) 10^{-2} cm^2 V^{-1} s^{-1} with a fixed hole mobility of 1 cm^2 V^{-1} s^{-1}. Simulations of dedoping with hole mobilities of (**D**) 10^{-1} cm^2 V^{-1} s^{-1}, (**E**) 1 cm^2 V^{-1} s^{-1}, and (**F**) 10 cm^2 V^{-1} s^{-1} with a fixed cation mobility of 10^{-3} cm^2 V^{-1} s^{-1}. Plot of dedoping time, taken as the time for the full length of the film to reach a differential transmission of $\Delta T/T_0 = -0.5$, for (**G**) varied cation mobilities and (**H**) varied hole mobilities. We find an inverse relationship between dedoping time and cation mobility and no dependence between dedoping time and hole mobility for the mobility values tested here.

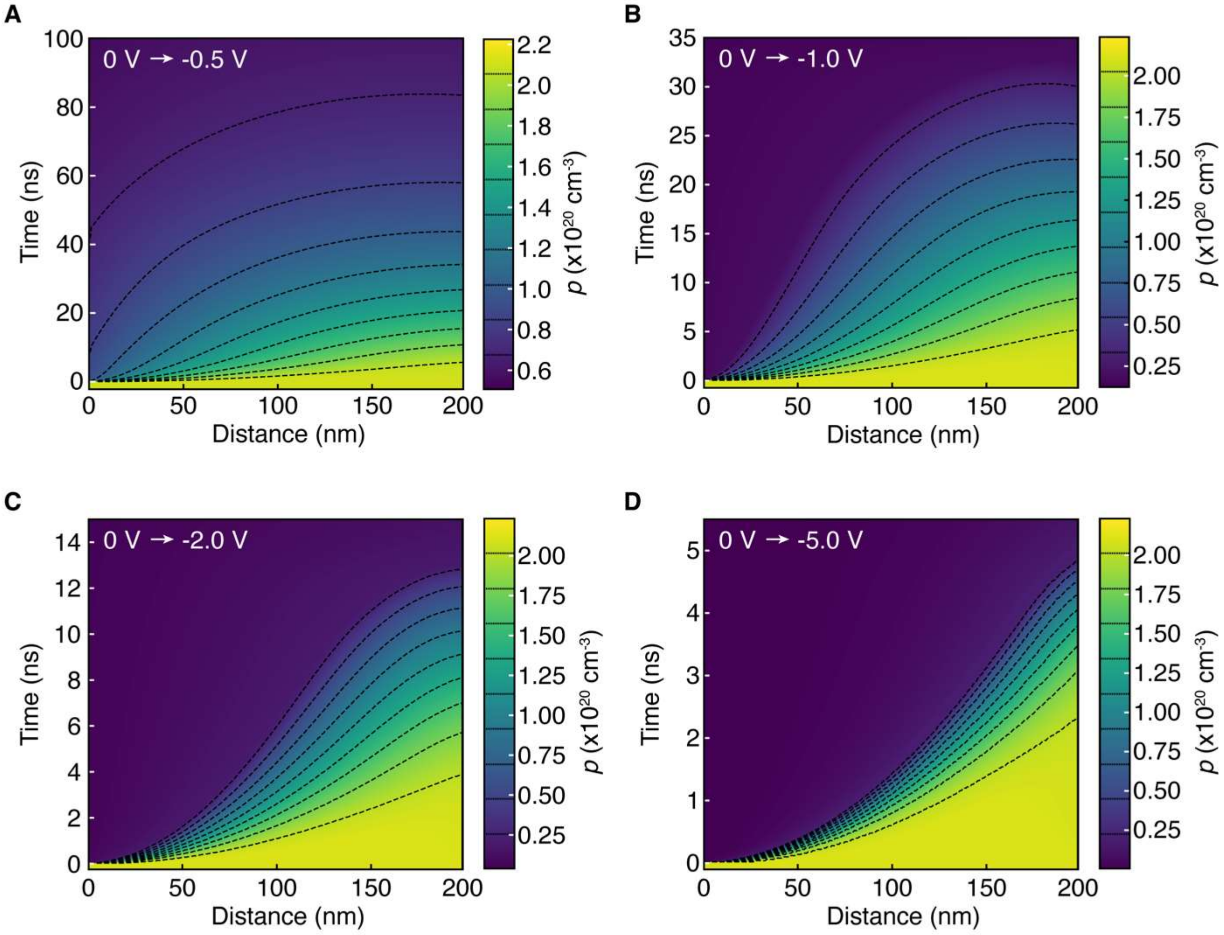

Fig. S7. Quasi-field drift-diffusion simulations with increasing driving potential.

Quasi-field drift diffusion simulations of dedoping with a driving potential (V_{WE}) of (**A**) -0.5 V, (**B**) -1.0 V, (**C**) -2.0 V, and (**D**) -5.0 V *vs.* Ag/AgCl. As the driving potential is increased, the qualitative behavior of dedoping shifts from diffusion-like (**Fig. S1**) to drift like (**Fig. 4C**).

Table S1. List of fit results for chronoamperometry.

**Dedoping**						
V_{dedope} **(mV)**	I_0 **(A)**	I_1 **(A)**	I_2 **(A)**	τ_1 **(s)**	τ_2 **(s)**	R^2
-800	-3.34×10^{-7}	-2.39×10^{-7}	-7.71×10^{-8}	0.138	2.74	0.986
-700	-2.72×10^{-7}	-1.97×10^{-7}	-6.20×10^{-8}	0.141	2.92	0.983
-600	-2.16×10^{-7}	-1.58×10^{-7}	-4.91×10^{-8}	0.149	3.21	0.982
-500	-1.73×10^{-7}	-1.27×10^{-7}	-3.93×10^{-8}	0.145	3.20	0.970
-400	-1.33×10^{-7}	-9.87×10^{-8}	-3.05×10^{-8}	0.146	3.27	0.959
-300	-9.46×10^{-8}	-7.11×10^{-8}	-2.19×10^{-8}	0.156	3.81	0.933
**Doping**						
$V_{i,dope}$ **(mV)**	I_0 **(A)**	I_1 **(A)**	I_2 **(A)**	τ_1 **(s)**	τ_2 **(s)**	R^2
-800	2.73×10^{-7}	1.90×10^{-7}	8.17×10^{-8}	0.209	3.20	0.990
-700	2.33×10^{-7}	1.70×10^{-7}	6.22×10^{-8}	0.207	2.55	0.988
-600	1.88×10^{-7}	1.39×10^{-7}	4.79×10^{-8}	0.215	3.86	0.984
-500	1.46×10^{-7}	1.08×10^{-7}	3.68×10^{-8}	0.223	3.91	0.974
-400	1.08×10^{-7}	7.95×10^{-8}	2.76×10^{-8}	0.227	4.02	0.959
-300	7.67×10^{-8}	5.68×10^{-8}	1.95×10^{-8}	0.235	4.29	0.931

Table S2. List of simulation parameters.

Physical parameter	Value used in simulation
Cation mobility	1×10^{-3} cm^2 V^{-1} s^{-1}
Sulfonate (anion) site density	2.1×10^{21} cm^{-3}
Initial hole concentration	2.8×10^{20} cm^{-3}
Initial cation concentration	1.82×10^{21} cm^{-3}
Volumetric capacitance	40 F cm^{-3}
Relative permittivity	80
Number of discrete intervals along channel	320
Number of discrete intervals in the electrolyte	80
Time per simulation step	1×10^{-17} s to 1×10^{-15} s
Electrolyte cation and anion concentration	6.022×10^{19} cm^{-3}
Ion mobility in the electrolyte*	1 cm^2 V^{-1} s^{-1}

*The ion mobility in the electrolyte is increased to account for the larger cross-sectional area for ion motion in the electrolyte compared to ions in the OMIEC channel.

REFERENCES AND NOTES

1. A. Sood, A. D. Poletayev, D. A. Cogswell, P. M. Csernica, J. T. Mefford, D. Fraggedakis, M. F. Toney, A. M. Lindenberg, M. Z. Bazant, W. C. Chueh, Electrochemical ion insertion from the atomic to the device scale. *Nat. Rev. Mater.* **6**, 847–867 (2021).

2. S. T. Keene, V. Gueskine, M. Berggren, G. G. Malliaras, K. Tybrandt, I. Zozoulenko, Exploiting mixed conducting polymers in organic and bioelectronic devices. *Phys. Chem. Chem. Phys.* **24**, 19144–19163 (2022).

3. S. L. Bidinger, S. T. Keene, S. Han, K. W. Plaxco, G. G. Malliaras, T. Hasan, Pulsed transistor operation enables miniaturization of electrochemical aptamer-based sensors. *Sci. Adv.* **8**, eadd4111 (2022).

4. S. T. Keene, D. Fogarty, R. Cooke, C. D. Casadevall, A. Salleo, O. Parlak, Wearable organic electrochemical transistor patch for multiplexed sensing of calcium and ammonium ions from human perspiration. *Adv. Healthc. Mater.* **8**, 1901321 (2019).

5. Y. van de Burgt, E. Lubberman, E. J. Fuller, S. T. Keene, G. C. Faria, S. Agarwal, M. J. Marinella, A. Alec Talin, A. Salleo, A non-volatile organic electrochemical device as a low-voltage artificial synapse for neuromorphic computing. *Nat. Mater.* **16**, 414–418 (2017).

6. A. A. Talin, Y. Li, D. A. Robinson, E. J. Fuller, S. Kumar, ECRAM materials, devices, circuits and architectures: A perspective. *Adv. Mater.* 2204771 (2022).

7. D. Moia, A. Giovannitti, A. A. Szumska, I. P. Maria, E. Rezasoltani, M. Sachs, M. Schnurr, P. R. F. Barnes, I. McCulloch, J. Nelson, Design and evaluation of conjugated polymers with polar side chains as electrode materials for electrochemical energy storage in aqueous electrolytes. *Energy Environ. Sci.* **12**, 1349–1357 (2019).

8. C. M. Proctor, J. Rivnay, G. G. Malliaras, Understanding volumetric capacitance in conducting polymers. *J. Polym. Sci. B* **54**, 1433–1436 (2016).

9. A. V. Volkov, K. Wijeratne, E. Mitraka, U. Ail, D. Zhao, K. Tybrandt, J. W. Andreasen, M. Berggren, X. Crispin, I. V. Zozoulenko, Understanding the capacitance of PEDOT:PSS. *Adv. Funct. Mater.* **27**, 1700329 (2017).

10. D. A. Bernards, G. G. Malliaras, Steady-state and transient behavior of organic electrochemical transistors. *Adv. Funct. Mater.* **17**, 3538–3544 (2007).

11. J. T. Friedlein, S. E. Shaheen, G. G. Malliaras, R. R. McLeod, Optical measurements revealing nonuniform hole mobility in organic electrochemical transistors. *Adv. Electron. Mater.* **1**, 1500189 (2015).

12. V. Kaphle, P. R. Paudel, D. Dahal, R. K. Radha Krishnan, B. Lüssem, Finding the equilibrium of organic electrochemical transistors. *Nat. Commun.* **11**, 2515 (2020).

13. K. Tybrandt, I. V. Zozoulenko, M. Berggren, Chemical potential–electric double layer coupling in conjugated polymer–polyelectrolyte blends. *Sci. Adv.* **3**, eaao3659 (2017).

14. B. D. Paulsen, K. Tybrandt, E. Stavrinidou, J. Rivnay, Organic mixed ionic–electronic conductors. *Nat. Mater.* **19**, 13–26 (2020).

15. J. Rivnay, S. Inal, B. A. Collins, M. Sessolo, E. Stavrinidou, X. Strakosas, C. Tassone, D. M. Delongchamp, G. G. Malliaras, Structural control of mixed ionic and electronic transport in conducting polymers. *Nat. Commun.* **7**, 11287 (2016).

16. S. T. Keene, W. Michaels, A. Melianas, T. J. Quill, E. J. Fuller, A. Giovannitti, I. McCulloch, A. A. Talin, C. J. Tassone, J. Qin, A. Troisi, A. Salleo, Efficient electronic tunneling governs transport in conducting polymer-insulator blends. *J. Am. Chem. Soc.* **144**, 10368–10376 (2022).

17. D. Tu, S. Fabiano, Mixed ion-electron transport in organic electrochemical transistors. *Appl. Phys. Lett.* **117**, 080501 (2020).

18. H. Kroemer, Quasi-electric fields and band offsets: Teaching electrons new tricks (Nobel Lecture). *ChemPhysChem* **2**, 490–499 (2001).

19. S. Yu, E. L. Ratcliff, Tuning organic electrochemical transistor (OECT) transconductance toward zero gate voltage in the faradaic mode. *ACS Appl. Mater. Interfaces* **13**, 50176–50186 (2021).

20. E. Stavrinidou, P. Leleux, H. Rajaona, D. Khodagholy, J. Rivnay, M. Lindau, S. Sanaur, G. G. Malliaras, Direct measurement of ion mobility in a conducting polymer. *Adv. Mater.* **25**, 4488–4493 (2013).

21. S. T. Keene, A. Melianas, E. J. Fuller, Y. van de Burgt, A. A. Talin, A. Salleo, Optimized pulsed write schemes improve linearity and write speed for low-power organic neuromorphic devices. *J. Phys. D Appl. Phys.* **51**, 224002 (2018).

22. E. J. Fuller, S. T. Keene, A. Melianas, Z. Wang, S. Agarwal, Y. Li, Y. Tuchman, C. D. James, M. J. Marinella, J. J. Yang, A. Salleo, A. A. Talin, Parallel programming of an ionic floating-gate memory array for scalable neuromorphic computing. *Science* **364**, 570–574 (2019).

23. J. D. Slinker, J. A. DeFranco, M. J. Jaquith, W. R. Silveira, Y.W. Zhong, J. M. Moran-Mirabal, H. G. Craighead, H. D. Abruña, J. A. Marohn, G. G. Malliaras, Direct measurement of the electric-field distribution in a light-emitting electrochemical cell. *Nat. Mater.* **6**, 894–899 (2007).

24. J. C. Demello, N. Tessler, S. C. Graham, R. H. Friend, Ionic space-charge effects in polymer light-emitting diodes. *Phys. Rev. B* **57**, 12951–12963 (1998).

25. S. van Reenen, P. Matyba, A. Dzwilewski, R. A. J. Janssen, L. Edman, M. Kemerink, A unifying model for the operation of light-emitting electrochemical cells. *J. Am. Chem. Soc.* **132**, 13776–13781 (2010).

26. S. Inal, G. G. Malliaras, J. Rivnay, Optical study of electrochromic moving fronts for the investigation of ion transport in conducting polymers. *J. Mater. Chem. C* **4**, 3942–3947 (2016).

27. G. Rebetez, O. Bardagot, J. Affolter, J. Réhault, N. Banerji, What drives the kinetics and doping level in the electrochemical reactions of PEDOT:PSS? *Adv. Funct. Mater.* **32**, 2105821 (2022).

28. R. Wu, B. D. Paulsen, Q. Ma, J. Rivnay, Mass and charge transport kinetics in an organic mixed ionic–electronic conductor. *Chem. Mater.* **34**, 9699–9710 (2022).

29. E. Stavrinidou, P. Leleux, H. Rajaona, M. Fiocchi, S. Sanaur, G. G. Malliaras, A simple model for ion injection and transport in conducting polymers. *J. Appl. Phys.* **113**, 244501 (2013).

30. C. G. Bischak, L. Q. Flagg, D. S. Ginger, Ion exchange gels allow organic electrochemical transistor operation with hydrophobic polymers in aqueous solution. *Adv. Mater.* **32**, 2002610 (2020).

31. S. Han, S. Yamamoto, A. G. Polyravas, G. G. Malliaras, Microfabricated ion-selective transistors with fast and super-nernstian response. *Adv. Mater.* **32**, 2004790 (2020).

32. Y. Zhang, M. Nguyen, C. Schnedermann, S. T. Keene, I. Jacobs, A. Rao, H. Sirringhaus, Transmission-based charge modulation microscopy on conjugated polymer blend field-effect transistors. *J. Chem. Phys.* **158**, 034201 (2023).

33. A. Giovannitti, R. B. Rashid, Q. Thiburce, B. D. Paulsen, C. Cendra, K. Thorley, D. Moia, J. T. Mefford, D. Hanifi, D. Weiyuan, M. Moser, A. Salleo, J. Nelson, I. McCulloch, J. Rivnay, Energetic control of redox-active polymers toward safe organic bioelectronic materials. *Adv. Mater.* **32**, 1908047 (2020).

34. C. Bonfil, Carleton University, (2014).

35. H. Bässler, D. Kroh, F. Schauer, V. Nádaždy, A. Köhler, Mapping the density of states distribution of organic semiconductors by employing energy resolved–electrochemical impedance spectroscopy. *Adv. Funct. Mater.* **31**, 2007738 (2021).

36. A. M. Nardes, M. Kemerink, R. A. J. Janssen, J. A. M. Bastiaansen, N. M. M. Kiggen, B. M. W. Langeveld, A. J. J. M. van Breemen, M. M. de Kok, Microscopic understanding of the anisotropic conductivity of PEDOT:PSS thin films. *Adv. Mater.* **19**, 1196–1200 (2007).

37. J. T. Friedlein, J. Rivnay, D. H. Dunlap, I. McCulloch, S. E. Shaheen, R. R. McLeod, G. G. Malliaras, Influence of disorder on transfer characteristics of organic electrochemical transistors. *Appl. Phys. Lett.* **111**, 023301 (2017).

38. J. Rivnay, P. Leleux, M. Sessolo, D. Khodagholy, T. Hervé, M. Fiocchi, G. G. Malliaras, Organic electrochemical transistors with maximum transconductance at zero gate bias. *Adv. Mater.* **25**, 7010–7014 (2013).

39. D. Ohayon, V. Druet, S. Inal, A guide for the characterization of organic electrochemical transistors and channel materials. *Chem. Soc. Rev.* **52**, 1001–1023 (2023).

40. B. D. Paulsen, R. Wu, C. J. Takacs, H.G. Steinrück, J. Strzalka, Q. Zhang, M. F. Toney, J. Rivnay, Time-resolved structural kinetics of an organic mixed ionic–electronic conductor. *Adv. Mater.* **32**, e2003404 (2020).

41. V. Jain, H. M. Yochum, R. Montazami, J. R. Heflin, Millisecond switching in solid state electrochromic polymer devices fabricated from ionic self-assembled multilayers. *Appl. Phys. Lett.* **92**, 033304 (2008).

42. J. Y. Kim, J.-Y. Oh, S. Cheon, H. Lee, J. Lee, J.-I. Lee, H. Ryu, S. M. Cho, T.-Y. Kim, C.-S. Ah, Y.-H. Kim, C.-S. Hwang, Optimized ion diffusion depth for maximizing optical contrast of environmentally friendly PEDOT:PSS electrochromic devices. *Opt. Mater. Express* **6**, 3127–3134 (2016).

43. I. Sahalianov, S. K. Singh, K. Tybrandt, M. Berggren, I. Zozoulenko, The intrinsic volumetric capacitance of conducting polymers: Pseudo-capacitors or double-layer supercapacitors? *RSC Adv.* **9**, 42498–42508 (2019).

44. P. Shiri, E. J. S. Dacanay, B. Hagen, L. G. Kaake, Vogel–Tammann–Fulcher model for charging dynamics in an organic electrochemical transistor. *J. Mater. Chem. C* **7**, 12935–12941 (2019).

45. M. Cucchi, A. Weissbach, L. M. Bongartz, R. Kantelberg, H. Tseng, H. Kleemann, K. Leo, Thermodynamics of organic electrochemical transistors. *Nat. Commun.* **13**, 4514 (2022).

46. X. Wang, X. Li, J. Mei, K. Zhao, Doping kinetics in organic mixed ionic–electronic conductors: Moving front experiments and the stress effect. *Extreme Mech. Lett.* **54**, 101739 (2022).

47. A. Khot, B. M. Savoie, How side-chain hydrophilicity modulates morphology and charge transport in mixed conducting polymers. *J. Polym. Sci.* **60**, 610–620 (2022).

48. L. Q. Flagg, L. E. Asselta, N. D'Antona, T. Nicolini, N. Stingelin, J. W. Onorato, C. K. Luscombe, R. Li, L. J. Richter, In situ studies of the swelling by an electrolyte in electrochemical doping of ethylene glycol-substituted polythiophene. *ACS Appl. Mater. Interfaces* **14**, 29052–29060 (2022).

49. V. Kaphle, S. Liu, A. Al-Shadeedi, C.-M. Keum, B. Lüssem, Contact resistance effects in highly doped organic electrochemical transistors. *Adv. Mater.* **28**, 8766–8770 (2016).

50. Y. Cao, G. Yu, A. J. Heeger, C. Y. Yang, Efficient, fast response light-emitting electrochemical cells: Electroluminescent and solid electrolyte polymers with interpenetrating network morphology. *Appl. Phys. Lett.* **68**, 3218–3220 (1996).

51. D. B. Riley, O. J. Sandberg, N. M. Wilson, W. Li, S. Zeiske, N. Zarrabi, P. Meredith, R. Österbacka, A. Armin, Direct quantification of quasi-fermi-level splitting in organic semiconductor devices. *Phys. Rev. Appl.* **15**, 064035 (2021).

52. P. Caprioglio, M. Stolterfoht, C. M. Wolff, T. Unold, B. Rech, S. Albrecht, D. Neher, On the relation between the open-circuit voltage and quasi-fermi level splitting in efficient perovskite solar cells. *Adv. Energy Mater.* **9**, 1901631 (2019).

53. A. Weigel, A. Sebesta, P. Kukura, Dark field microspectroscopy with single molecule fluorescence sensitivity. *ACS Photonics* **1**, 848–856 (2014).

54. C. Bonafil, Carleton University, Carleton University (2014).

55. G. LeCroy, C. Cendra, T. J. Quill, M. Moser, R. Hallani, J. F. Ponder, K. Stone, S. D. Kang, A. Y.-L. Liang, Q. Thiburce, I. McCulloch, F. C. Spano, A. Giovannitti, A. Salleo, Role of aggregates and microstructure of mixed-ionic–electronic-conductors on charge transport in electrochemical transistors. *Mater. Horiz.* **10**, 2568–2578 (2023).
